# Diagnosing migraine from genome-wide genotype data: a machine learning analysis

**DOI:** 10.1093/brain/awaf172

**Published:** 2025-05-06

**Authors:** Antonios Danelakis, Tjaša Kumelj, Bendik S Winsvold, Marte Helene Bjørk, Parashkev Nachev, Manjit Matharu, Dominic Giles, Erling Tronvik, Helge Langseth, Anker Stubberud, The International Headache Genetics Consortium, Verneri Anttila, Ville Artto, Andrea C Belin, Anna Bjornsdottir, Gyda Bjornsdottir, Dorret I Boomsma, Sigrid Børte, Mona A Chalmer, Daniel I Chasman, Bru Cormand, Ester Cuenca-Leon, George Davey-Smith, Irene de Boer, Martin Dichgans, Tonu Esko, Tobias Freilinger, Padhraig Gormley, Lyn R Griffiths, Eija Hämäläinen, Thomas F Hansen, Aster V E Harder, Heidi Hautakangas, Marjo Hiekkala, Maria G Hrafnsdottir, M Arfan Ikram, Marjo-Riitta Järvelin, Risto Kajanne, Mikko Kallela, Jaakko Kaprio, Mari Kaunisto, Lisette J A Kogelman, Espen S Kristoffersen, Christian Kubisch, Mitja Kurki, Tobias Kurth, Lenore Launer, Terho Lehtimäki, Davor Lessel, Lannie Ligthart, Sigurdur H Magnusson, Rainer Malik, Bertram Müller-Myhsok, Carrie Northover, Dale R Nyholt, Jes Olesen, Aarno Palotie, Priit Palta, Linda M Pedersen, Nancy Pedersen, Matti Pirinen, Danielle Posthuma, Patricia Pozo-Rosich, Alice Pressman, Olli Raitakari, Caroline Ran, Gudrun R Sigurdardottir, Hreinn Stefansson, Kari Stefansson, Olafur A Sveinsson, Gisela M Terwindt, Thorgeir E Thorgeirsson, Arn M J M van den Maagdenberg, Cornelia van Duijn, Maija Wessman, Bendik S Winsvold, John-Anker Zwart

**Affiliations:** NorHead Norwegian Centre for Headache Research, NTNU Norwegian University of Science and Technology, Trondheim 7030, Norway; Department of Computer Science, NTNU Norwegian University of Science and Technology, Trondheim 7034, Norway; NorHead Norwegian Centre for Headache Research, NTNU Norwegian University of Science and Technology, Trondheim 7030, Norway; Department of Neuromedicine and Movement Science, NTNU Norwegian University of Science and Technology, Trondheim 7030, Norway; NorHead Norwegian Centre for Headache Research, NTNU Norwegian University of Science and Technology, Trondheim 7030, Norway; Department of Research and Innovation, Division of Clinical Neuroscience, Oslo University Hospital, Oslo 0450, Norway; Department of Neurology, Oslo University Hospital, Oslo 0372, Norway; HUNT Center for Molecular and Clinical Epidemiology, Department of Public Health and Nursing, NTNU Norwegian University of Science and Technology, Trondheim 7030, Norway; NorHead Norwegian Centre for Headache Research, NTNU Norwegian University of Science and Technology, Trondheim 7030, Norway; Department of Clinical Medicine, University of Bergen, Bergen 5021, Norway; Department of Neurology, Haukeland University Hospital, Bergen 5053, Norway; High Dimensional Neurology Group, UCL Institute of Neurology, University College London, London WC1N 3BG, UK; NorHead Norwegian Centre for Headache Research, NTNU Norwegian University of Science and Technology, Trondheim 7030, Norway; Headache and Facial Pain Group, UCL Institute of Neurology and National Hospital for Neurology and Neurosurgery, London WC1N 3BG, UK; High Dimensional Neurology Group, UCL Institute of Neurology, University College London, London WC1N 3BG, UK; NorHead Norwegian Centre for Headache Research, NTNU Norwegian University of Science and Technology, Trondheim 7030, Norway; Department of Neuromedicine and Movement Science, NTNU Norwegian University of Science and Technology, Trondheim 7030, Norway; Neuroclinic, St Olav University Hospital, Trondheim 7030, Norway; NorHead Norwegian Centre for Headache Research, NTNU Norwegian University of Science and Technology, Trondheim 7030, Norway; Department of Computer Science, NTNU Norwegian University of Science and Technology, Trondheim 7034, Norway; NorHead Norwegian Centre for Headache Research, NTNU Norwegian University of Science and Technology, Trondheim 7030, Norway; Department of Neuromedicine and Movement Science, NTNU Norwegian University of Science and Technology, Trondheim 7030, Norway

**Keywords:** artificial intelligence, gradient boosting, headache, genetics, epistasis, HUNT

## Abstract

Migraine has an assumed polygenic basis, but the genetic risk variants identified in genome-wide association studies only explain a proportion of the heritability. We aimed to develop machine learning models, capturing non-additive and interactive effects, to address the missing heritability.

This was a cross-sectional population-based study of participants in the second and third Trøndelag Health Study. Individuals underwent genome-wide genotyping and were phenotyped based on validated modified criteria of the International Classification of Headache Disorders. Four datasets of increasing numbers of genetic variants were created using different thresholds of linkage disequilibrium and univariate genome-wide associated *P*-values. A series of machine learning and deep learning methods were optimized and evaluated. The genotype tools PLINK and LDPred2 were used for polygenic risk scoring. Models were trained on a partition of the dataset and tested in a hold-out set. The area under the receiver operating characteristics curve was used as the primary scoring metric. Classification by machine learning was statistically compared to that of polygenic risk scoring. Finally, we explored the biological functions of the variants unique to the machine learning approach.

Overall, 43 197 individuals (51% women), with a mean age of 54.6 years, were included in the modelling. A light gradient boosting machine performed best for the three smallest datasets (108, 7771 and 7840 variants), all with hold-out test set area under curve at 0.63. A multinomial naïve Bayes model performed best in the largest dataset (140 467 variants) with a hold-out test set area under curve of 0.62. The models were statistically significantly superior to polygenic risk scoring (area under curve 0.52 to 0.59) for all the datasets (*P* < 0.001 to *P* = 0.02). Machine learning identified many of the same genes and pathways identified in genome-wide association studies, but also several unique pathways, mainly related to signal transduction and neurological function. Interestingly, pathways related to botulinum toxins, and pathways related to the calcitonin gene-related peptide receptor also emerged.

This study suggests that migraine may follow a non-additive and interactive genetic causal structure, potentially best captured by complex machine learning models. Such structure may be concealed where the data dimensionality (high number of genetic variants) is insufficiently supported by the scale of available data, leaving a misleading impression of purely additive effects. Future machine learning models using substantially larger sample sizes could harness both the additive and the interactive effects, enhancing precision and offering deeper understanding of genetic interactions underlying migraine.

## Teaser

Danelakis *et al.* show that machine learning outperforms polygenic risk scoring in identifying individuals with migraine from genome-wide genotype data. These findings support a non-additive genetic architecture for migraine and may provide insights into its underlying biological mechanisms.

## Introduction

Migraine is a common primary headache disorder with a substantial global disease burden.^1^ The global prevalence is estimated to 14%,^2^ and it is ranked second among causes of disability, and first among women under 50 years of age.^3^ Migraine is characterized by recurring attacks of intense, often unilateral and pulsating headaches, accompanied by nausea, vomiting, and sensitivity to light and sound.^4^ In up to a third of individuals the attacks are at times preceded by transient focal neurological aura symptoms, most commonly visual or sensory.

The aetiology of migraine is complex and incompletely understood. Inheritance has long been recognized as important, as migraine tends to cluster in families.^5,6^ Twin studies have confirmed consistently higher concordance rates of migraine in monozygotic twins versus dizygotic twins,^7^ with an estimated heritability of around 50%.^8^

The largest genome-wide association study (GWAS) meta-analysis identified 123 migraine risk loci.^9^ Other GWAS have identified similar and other risk variants.^10^  ^,11^ Yet, the sum of the risk variants does not explain the full heritability of migraine.^12^ Indeed, it was estimated that the 123 risk loci from the 2022 GWAS only explain 11.2% of the heritability. The missing heritability—defined as the gap between heritability estimates from twin studies and the heritability explained by the identified genetic variants—may be attributable to at least two factors.^13^ First, there are likely many small-effect-variants that increase the risk of migraine but fail to reach the significance level required in GWAS. Second, there may be epistatic interactions, where the effect of one gene is modified by other genes complicating the genetic architecture. This results in an overall effect that is not merely the sum of each gene’s contribution (additive effects) but is instead driven by the combined influence of interacting genes (non-additive effects).^14^

Polygenic risk scoring (PRS) can be used to estimate the additive risk of complex traits based on the sum of all risk alleles carried by an individual.^15^ This summing across variants assumes an additive genetic architecture, with independence of risk variants,^15^ and does not take into account any gene-gene or gene-environment interactions.^16^ Such an approach is not suited to explain any interactive genetic factors contributing to the missing heritability.

Therefore, implementing a model that accounts for interactive effects—in addition to additive effects—could distinguish individuals with migraine from headache-free controls using genotype data with better precision. Importantly, it could also help explain the missing heritability and increase our understanding of the genetic architecture of migraine. We hypothesized that complex, high-dimensional machine learning models that can handle a large number of input variables while preserving covariate interactions may address the shortcomings of PRS.

The objectives of this study were to (i) estimate the accuracy of machine learning in distinguishing migraine from genome-wide genotype data; (ii) compare the diagnostic accuracy of machine learning models with PRS across increasing dimensionalities (increasing number of genetic variants) of genetic input data; and (iii) evaluate possible biological mechanisms of genes and interactions identified through machine learning modelling.

## Materials and methods

### Data sources and data materials

This was a cross-sectional population-based machine learning analysis of genome-wide genotype data for classifying individuals with migraine versus headache-free controls. The methods for acquiring genotype data and phenotype assignment were similar to those reported in previous studies of the same health survey and biobank data material.^17,18^

#### The Trøndelag health study

The Trøndelag Health Study (HUNT) is a large, population-based cohort study from Trøndelag county in Norway that has been carried out in four waves (HUNT1 to HUNT4).^19^ All inhabitants aged 20 years or older living in the county were invited to participate. Participation was based on informed, written consent, and the study was approved by the Regional Committee for Medical and Health Research (#2015/576/REK Midt and #2014/144/REK Midt). Data were collected through questionnaires and clinical examinations. DNA from whole blood was collected in HUNT2 (1995–1997) and HUNT3 (2006–2008). Questionnaire data for phenotype assignment were collected in HUNT2 and HUNT3.

#### Genotyping

Genotyping of HUNT2 and HUNT3 participants was performed at the Genomics-Core Facility at the NTNU Norwegian University of Science and Technology. Three different versions of the Illumina HumanCoreExome microarray (Illumina HumanCoreExome12 v.1.0, HumanCoreExome12 v.1.1 and HumanCoreExome24 with custom content) were used. The quality control and imputation has been described in detail elsewhere.^20^ In brief, after rigorous quality control, genotypes were imputed using a customized reference panel consisting of the Haplotype Reference consortium release 1.1. Finally, variants with imputation quality *r*^2^ < 0.3 were excluded.

#### Phenotype assignment

A diagnosis of migraine was assessed using a modified version of the International Classification of Headache Disorders^21,22^ based on questionnaires in HUNT2 and HUNT3. Participants were asked whether they had suffered from headache during the last 12 months, and those who answered ‘yes’ were classified as headache sufferers, while those who answered ‘no’ constituted the control group of headache-free individuals. Those answering ‘yes’ were subsequently asked questions about their headache to assess whether they fulfilled criteria for migraine or not. Those fulfilling the criteria for migraine were classified as migraine cases in this study. This method of phenotype assignment is reported in detail elsewhere and has been validated through clinical interviews by a headache neurologist.^23,24^

#### Genome-wide association study data and dataset creation

From the largest migraine GWAS meta-analysis, which was based on 102 084 cases with migraine and 771 257 controls,^9^ we acquired summary statistics for the lead variants of the 123 identified migraine risk loci, and the 8117 migraine variants that reached the genome-wide significance threshold of *P* < 5 × 10^−8^. Because HUNT participants were part of the GWAS meta-analysis,^9^ a reverse meta-analysis was conducted to derive a new beta coefficient and standard error for each variant after excluding individuals from the HUNT study. Using the recalculated beta and standard error, updated *P*-values for the migraine association were obtained by calculating the cumulative density function of a normal distribution, with a mean of 0.0 and a standard deviation of 1.0. This method of reverse meta-analysis allowed us to re-calculate the summary statistics without being influenced by HUNT individuals, in turn allowing machine learning and PRS classification of the ‘unseen’ HUNT samples. The re-calculated summary statistics were used to create Datasets 1 and 2 (see later).

In addition to the 2022 GWAS meta-analysis^9^ summary statistics for significant variants, we used the complete, genome-wide summary statistics from this meta-analysis after excluding 23andMe, owing to data availability.^9^ A reverse meta-analysis method, as described above, was again used to remove the influence of the HUNT individuals. These summary statistics were used to create Datasets 3 and 4 (see later).

To compare the diagnostic accuracy of machine learning versus PRS and evaluate the effect of the dimensionality of the genotype input data, four datasets containing increasing numbers of genetic variants were created as follows:

1. the linkage disequilibrium independent variants with an *r*^2^ threshold of 0.1, reaching the genome-wide significance threshold of *P* < 5 × 10^−8^, identified among the 8117 genome-wide significant variants from the 2022 GWAS meta-analysis^9^
  after having performed reverse meta-analysis to remove HUNT individuals;
2. the variants available in our dataset, among the 8117 variants from the 2022 GWAS meta-analysis,^9^ reaching the genome-wide significance level of *P* < 5 × 10^−8^ after having performed reverse meta-analysis to remove HUNT individuals;
3. the variants reaching a significance level of *P* < 1 × 10^−5^ captured from the summary statistics of the 2022 GWAS meta-analysis^9^ excluding 23andMe and after having performed reverse meta-analysis to remove HUNT individuals;
4. the linkage disequilibrium-independent variants, at an *r*^2^ threshold of 0.1, from available variants in the summary statistics of the 2022 GWAS meta-analysis^9^ excluding 23andMe and after having performed reverse meta-analysis to remove HUNT individuals.

### Machine diagnostic modelling

**Figure 1:**
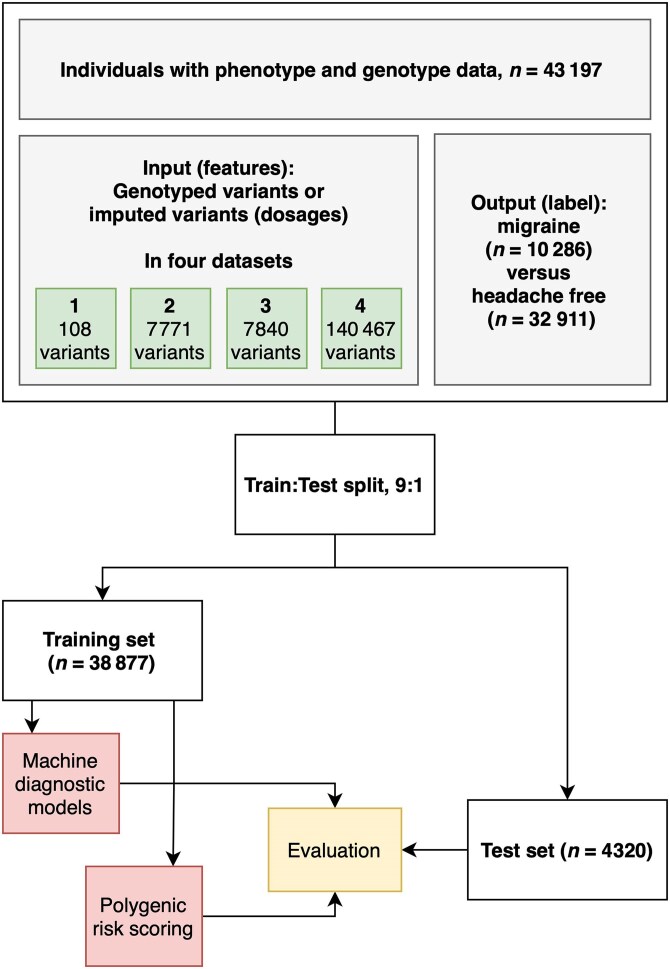
**Schematic overview of the study design.** Among 43 197 individuals, 10 286 had migraine and 32 911 were headache-free controls. Four different datasets with an increasing number of genetic variants were used for distinguishing migraine versus headache-free controls. These datasets were split in the same 9:1 ratio training and test sets. The training data were subsequently preprocessed, trained and optimized using 10-fold cross-validation. The best model for each dataset was evaluated on the test set.

The genotyped variants (where available) or imputed variants (dosages, i.e. a decimal number between 0 and 2 describing the probability of the imputation corresponding to a given allele combination) were used as input variables (features) for the models. The genetic variant dosages were one-hot-encoded (redefined as dummy variables) in Datasets 1–3. Dataset 4 was not one-hot-encoded because its dimensionality (number of variables) was already significant and one-hot-encoding would 3-fold the feature size, resulting in a problematically large feature-to-sample size ratio.^25^ The phenotype assignment (migraine or headache-free) was used as the outcome (label). Figure 1 is a schematic of the study design and modelling strategy.

The data were split in a random stratified fashion into a training and a test set in a 9:1 ratio. The test set was the same for all machine learning and PRS models and was kept unseen until the final model evaluation.

A series of standard machine learning classification architectures were evaluated: logistic regression; least absolute shrinkage and selection operator; support vector machines; decision trees; k-nearest neighbours; naïve Bayes; random forest; gradient boosting methods; and ensemble methods. Owing to the substantial number of features for Dataset 4, these data were trained in chunks with the following classifiers: perceptron; stochastic gradient descent; passive-aggressive classifier; and multinomial, Bernoulli, Gaussian and complement naïve Bayes. Finally, deep learning architectures, including TabNet,^26^ GenNet^27^ and FDDN^28^ (specifically to tackle the issue with input dimensionality surpassing sample size), were evaluated. GenNet is a deep learning network that preprocesses data based on genetic annotation. FDNN is a deep learning network specifically developed to handle cases with a large feature-to-sample ratio.

Model hyperparameters were optimized using Optuna^29^ with 500 trials. Models were trained on the training set and performance was continuously evaluated with 10-fold cross-validation. For the largest dataset a single train/validate split was used owing to extensive compute time. The area under the receiver operating characteristics curve (AUC) was used as a scoring metric for training and optimizing the models. The mean AUC and its standard deviation (SD) were calculated across the 10 training folds to summarize the model’s training performance and the variability between folds. In addition to AUC, we calculated accuracy, precision, recall and F1-score. The precision is the proportion of those classified as migraine that indeed have migraine and is identical to the positive predictive value. The recall is the proportion of those that have migraine that were classified as migraine and is the same as sensitivity. The F1-score is a compound metric of precision and recall. The top performing model for each of the four datasets was finally applied on the test set to quantify out-of-sample performance, calculating AUC with 95% confidence intervals (CI). All machine learning analyses were conducted using Python 3.10 (Python Software Foundation) with open-source packages (Supplementary Table 1).

Sample characteristics, demographics and phenotype assignment were statistically described as proportions for dichotomous variables and means with SD for continuous variables.

#### Sensitivity analysis of relatedness

To estimate the influence of relatedness of individuals, we conducted a sensitivity analysis on unrelated individuals (up to 3rd degree relatedness was clumped), using the PLINK command *plink2 –bfile plinkFileName –king-cutoff 0.006*.

#### Sensitivity analysis of feature dimensionality

*Post hoc*, a series of intermediate datasets with a number of variants between Dataset 3 and Dataset 4 was created to better elucidate the impact of feature dimensionality and model complexity. These datasets were created by changing the linkage disequilibrium threshold. Each of the intermediate datasets was used to train the best simple additive machine learning model and the best complex machine learning model.

### Polygenic risk scoring

Two methods were employed for calculating PRS: PLINK,^30^ and LDpred2.^31^ PLINK extracts the PRS based on the clumping and thresholding approach. This minimizes the risk of overrepresenting certain genomic regions due to high linkage disequilibrium, using a linkage disequilibrium clumping correlation (*r*^2^) cut-off value of 0.1 to determine which variants are considered too correlated. PRS was calculated for each subject using five different *P*-value thresholds (10^−7^, 10^−8^, 10^−9^, 10^−10^ and 10^−11^) representing the significance of association with the migraine label. The *P*-value threshold with the best model fit was used. Population structure was accounted for by incorporating 10 principal components capturing ancestry-related differences as covariates to make the findings more reliable across diverse groups. Finally, the PRSs were normalized, and a 0.5 decision threshold was used to distinguish cases and controls. LDpred2 also performs linkage disequilibrium clumping and accounts for population stratification using 10 principal components, similar to PLINK. Thereafter, a logistic regression model is trained to achieve the best fit of the PRS on the data. Finally, the trained regression model is used to classify the phenotype. The dataset train/test split used for hold-out test set evaluation of the PRS approaches was identical to that of the machine learning models.

### Comparison of machine diagnostics with polygenic risk scoring

To compare the diagnostic performance of machine learning and PRS, the test set AUCs were compared statistically. The null hypothesis criterion was tested by performing the Wilcoxon non-parametric test of independent samples.^32^ The statistical significance threshold was set at 0.05.

### Model explainability

Using the top performing model, we constructed calibration plots to check how accurately the model’s classification matched the actual migraine outcomes. We also constructed probability density curves for both the machine learning models and PRS to visualize the separability of cases and controls. For the top performing machine learning model for each of the four datasets, we calculated Shapley values. For each dataset, variants were ordered by Shapley values from highest to lowest and compared to the GWAS meta-analysis.^9^ We also constructed SHAP (Shapley Additive exPlanations) summary plots to visualize the relative contribution of increasing genotype input dimensionality.^33^ SHAP is a framework utilizing Shapley values to explain machine learning model predictions. SHAP assigns each feature an importance value, which enables interpretation of how much the feature contributes towards the prediction.

### Gene annotation and pathway enrichment

To perform gene annotation and pathway enrichment analyses, we identified the most influential variants in the top performing machine learning models and the lead variants identified in the GWAS meta-analysis. For fair comparison, the 123 most important variants were selected. In cases where several variants were considered equally important, so that the total number exceeded 123, all those of equal importance were used. Feature importances were extracted from the models and prioritized by their importance, providing an ordered list of each variant’s contribution to the model.

Annotations of variants to genes were based on the proximity method that maps a genetic variant with its nearest gene (or to each of the genes it directly overlaps), using SNPnexus^34^ with EnsemblDB^35^ as a mapping reference.

Next, annotations of genes to pathways were performed using the Reactome Pathway Database,^36^ as implemented in SNPnexus. Pathway enrichment *P*-values were adjusted for multiple testing using the Benjamini-Hochberg method to control the false discovery rate.^36^ The crude significance threshold was set at 0.05, while the false discovery rate threshold was set to 0.1. The same annotation and pathway enrichment approach was used for both the variants identified through GWAS and the variants identified through machine learning.

## Results

### Sample characteristics

#### Demographics and phenotype assignment

In total, 43 197 individuals with available genotype and phenotype data were included in the analyses. Supplementary Fig. 1 is a flow-chart of the study population. Altogether, 10 286 individuals (24%) were classified as having migraine and 32 911 (76%) were classified as headache-free controls. Among those with migraine, 7225 (70%) were women, and among the headache-free controls 15 088 (46%) were women. The mean age of the overall population was 54.6 (SD = 17.3). The mean age of the migraine cases and the headache-free controls was 46.8 (SD = 14.0) and 57.1 (SD = 17.5), respectively. All participants were of European ancestry. The distributions of individuals with migraine and headache-free controls were similar in the training, validation and test splits. In the training set, 8322 (24%) had migraine and 26 667 (76%) were headache-free; in the validation set for Dataset 4, 925 (24%) had migraine and 2963 (76%) were headache-free; and in the test set, 1039 (24%) had migraine and 3281 (76%) were headache-free. Previous clinical validations of the phenotype assignment found that in HUNT2, the sensitivity was 69% and the specificity 89% for migraine.^23^ In HUNT3, the sensitivity was 67% and the specificity was 96%.^24^

#### Genotype data

In HUNT2 and HUNT3, 71 680 individuals were genotyped. After quality control and imputation, a total of 9 832 846 variants were available for the 43 197 individuals included in the analysis.

In the reverse meta-analysis procedure, the influence of 7801 cases and 32 423 controls from the HUNT study was removed in the creation of Datasets 1–4, and the influence of 53 109 cases and 230 876 controls from 23andMe was removed in the creation of Datasets 3 and 4. Thus 94 283 cases and 738 834 controls were used to calculate summary statistics for Datasets 1 and 2 and 41 174 cases and 507 958 controls were used to calculate summary statistics for Datasets 3 and 4. Of note, 1395 migraine cases and 1011 controls from HUNT could not be removed from the summary statistic calculation as they were part of a previous meta-analysis^37^ already included in the 2022 GWAS meta-analysis.^9^

After quality control, imputation, the reverse meta-analysis procedure, calculation of summary statistics, linkage disequilibrium pruning and pruning based on *P*-values for variant association, the number of variants in the four datasets were: Dataset 1: 108 variants; Dataset 2: 7771 variants; Dataset 3: 7840 variants; and Dataset 4: 140 467 variants.

### Machine diagnostic performance

For the first three datasets (108, 7771 and 7840 variants), the top performing model was the light gradient boosting machine classifier, with cross-validated AUCs between 0.64 and 0.65, and cross-validated accuracies between 0.60 and 0.62 (Table 1). The hold-out test set AUC was 0.63 (95% CI: 0.61–0.65), 0.63 (95% CI: 0.61–0.65) and 0.63 (95% CI: 0.62–0.66) for the datasets with 108, 7771 and 7804 variants, respectively. The corresponding hold-out test accuracies were 0.60, 0.60 and 0.61. The hold-out test set precision ranged from 0.59 to 0.60, recall ranged from 0.59 to 0.60, and the F1-score ranged from 0.55 to 0.57 (Table 1)

**Table 1:** Performance of best machine learning models

	AUC		Accuracy		Recall		Precision		F1-Score	
Dataset	Train	Test	Train	Test	Train	Test	Train	Test	Train	Test
Dataset 1 (108 variants)^a^	0.64 ± 0.010	0.63	0.60 ± 0.009	0.60	0.59 ± 0.010	0.59	0.59 ± 0.010	0.59	0.55 ± 0.009	0.55
Dataset 2 (7771 variants)^b^	0.64 ± 0.010	0.63	0.62 ± 0.008	0.61	0.60 ± 0.009	0.59	0.60 ± 0.009	0.59	0.55 ± 0.009	0.55
Dataset 3 (7840 variants)^c^	0.65 ± 0.012	0.63	0.61 ± 0.009	0.62	0.60 ± 0.011	0.60	0.60 ± 0.011	0.60	0.58 ± 0.010	0.57
Dataset 4 (140 467 variants)^d^	0.62	0.62	0.59	0.58	0.57	0.56	0.65	0.64	0.45	0.43

For each scoring metric, the training set performance is presented as the mean of 10-fold cross validation (± standard deviation), except for Dataset 4, in which only one train/validate split was evaluated. The test value is the performance of the trained model in the hold-out test set. AUC = area under the receiver operator characteristic curve.

^a^Lead variants from risk loci identified in the 2022 genome-wide association study meta-analysis^10^ (108 variants).

^b^All variants with *P*-value <5 × 10^−8^ identified from the 2022 genome-wide association study meta-analysis^10^ summary statistics (7771 variants).

^c^All variants with a *P*-value <1 × 10^−5^ identified from the re-calculated summary statistics without 23andMe (7840 variants).

^d^All linkage disequilibrium independent variants among all genotyped variants (140 467 variants).

**Figure 2:**
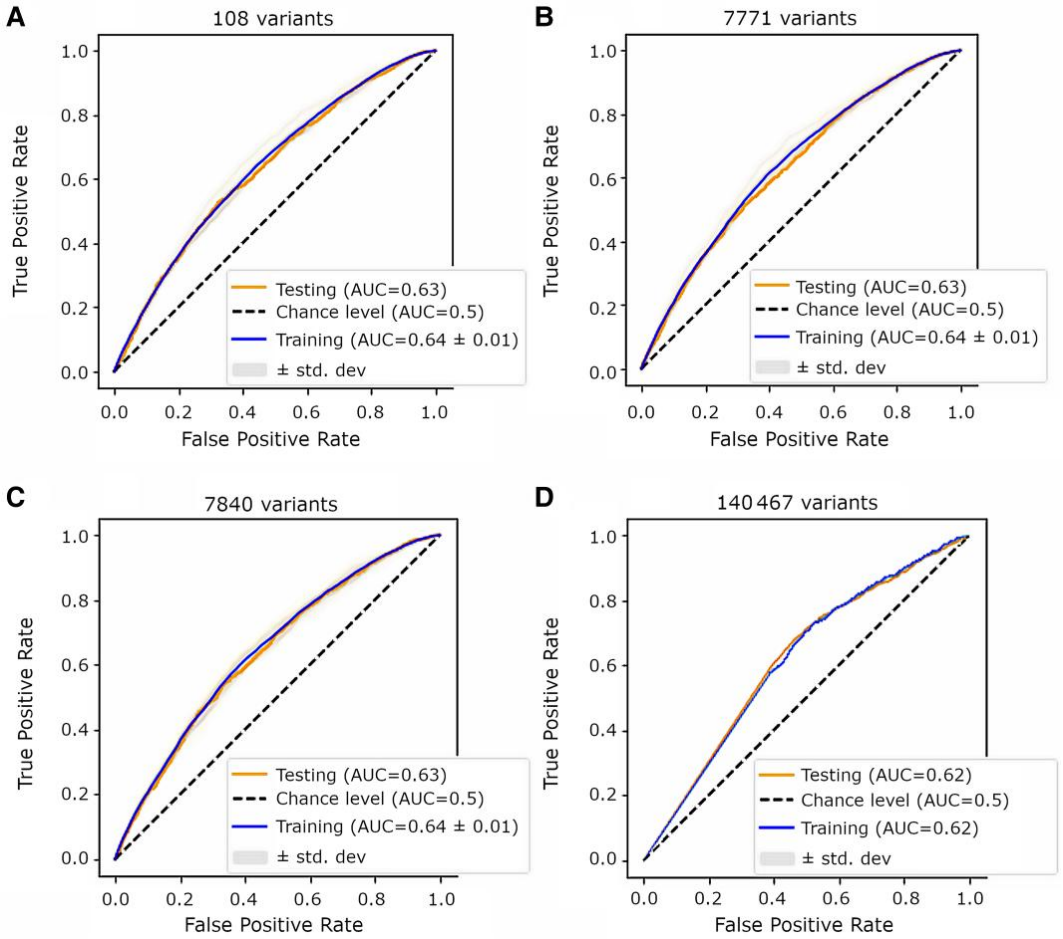
**Performance of the best machine learning models.** Receiver operating characteristics curves for top performing machine learning models for each of the four datasets showing mean 10-fold cross-validated area under curve (blue line) ± 1 standard deviation (grey shaded area), and test set area under curve (orange line). (**A**) Dataset 1 with 108 variants. (**B**) Dataset 2 with 7771 variants. (**C**) Dataset 4 with 7840 variants. (**D**) Dataset 4 with 140 467 variants.

In the largest dataset, containing 140 467 variants, the top performing model was the multinomial naïve Bayes classifier, which achieved a validation set AUC of 0.62 and an accuracy of 0.57. The hold-out test set AUC was 0.62 (95% CI: 0.60–0.64), and the accuracy was 0.58. The test set precision, recall and F1-score were 0.64, 0.56 and 0.43, respectively. Table 1 and Fig. 2 provide additional training and test performance metrics for the models. Supplementary Table 2 provides all the out-of-sample and training experimental results for the best models of each learning approach for every dataset.

#### Relatedness sensitivity analysis

Overall, 3567 migraine cases and 10 417 controls were unrelated and included in the relatedness sensitivity analysis. The mean cross-validated training AUC for the relatedness sensitivity analysis ranged from 0.62 to 0.63 for all four datasets. The corresponding test set AUCs ranged from 0.61 to 0.63. Supplementary Table 3 outlines all performance metrics for the sensitivity analysis.

#### Feature dimensionality sensitivity analysis

**Figure 3:**
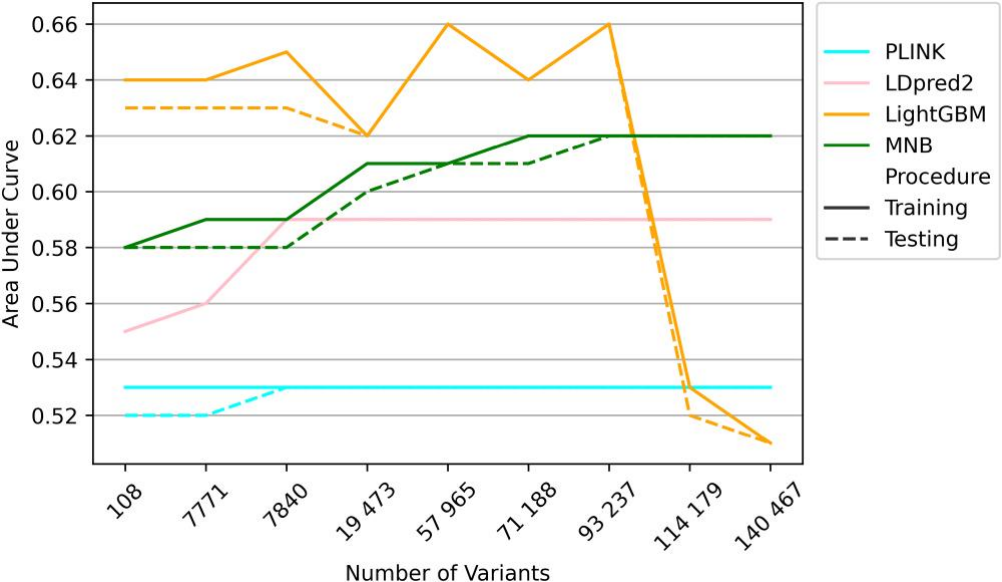
**Impact of feature dimensionality.** The hold-out test set area under curve (y-axis) is plotted against the number of variants included in the model (*y*-axis) for the best machine learning and polygenic risk scoring approaches. For each colour, solid lines represent training performance and dotted lines represent test performance for a given modelling approach. Performance for the intermediate datasets (19 473 to 114 179 variants) were only calculated for the best non-linear complex machine learning approach (light gradient boosting) and the best simple additive model (multinomial naïve Bayes) as part of the *post hoc* sensitivity analyses. Note that light gradient boosting increases in performance up to 93 237 variants before sharply dropping, indicating overfitting when the feature space exceeds a limit. Multinomial naïve Bayes, however, increases steadily before reaching a plateau beyond 57 965 variants. LightGBM = light gradient boosting machine. MNB = multnomial naïve Bayes.

Five intermediate datasets with 19 473, 57 965, 71 188, 93 237 and 114 179 variants were created. With increasing feature dimensionality (i.e. higher number of variants), the performance of the complex models increased until suddenly reaching a performance drop, whereas the performance of simpler additive models such as the multinomial naive Bayes increased steadily until reaching a plateau (Fig. 3 and Supplementary Table 2). The light gradient boosting machine peaked at 92 237 variants with a training and test set AUC of 0.66.

### Polygenic risk scores

The test set PRS AUCs using PLINK were 0.52 (95% CI 0.50–0.54) for the dataset with 108 variants, 0.52 (95% CI 0.51–0.55) for the dataset with 7771 variants, 0.53 (95% CI 0.51–0.55) for the dataset with 7840 variants, and 0.53 (95% CI 0.52–0.56) for the dataset with 140 467 variants. The corresponding test set PRS AUCs using LDpred2 were 0.55 (95% CI 0.53–0.57), 0.56 (95% CI 0.54–0.58), 0.59 (95% CI 0.57–0.61), and 0.59 (95% CI 0.57–0.61), respectively.

### Comparison of machine learning and polygenic risk scores

The machine learning models outperformed PRS in all four datasets (Table 2). The difference in AUCs was most pronounced for Datasets 1 through 3 (*P* < 0.001), and slightly less pronounced for the largest dataset (*P* = 0.02). Figure 3 illustrates the impact of feature dimensionality on performance for both machine learning and PRS.

**Table 2:** Comparison of machine learning and polygenic risk scoring

Dataset	Best machine learning model AUC (95% CI)	PLINK AUC (95% CI)	LDpred2 AUC (95% CI)	Comparison
Dataset 1 (108 variants)^a^	0.63 (0.61–0.65)	0.52 (0.50–0.54	0.55 (0.53–0.57)	*P* < 0.001
Dataset 2 (7771 variants)^b^	0.63 (0.61–0.65)	0.52 (0.51–0.55)	0.56 (0.54–0.58)	*P* < 0.001
Dataset 3 (7840 variants)^c^	0.63 (0.62–0.66)	0.53 (0.51–0.55)	0.59 (0.57–0.61)	*P* < 0.001
Dataset 4 (140 467 variants)^d^	0.62 (0.60–0.64)	0.53 (0.52–0.56)	0.59 (0.57–0.61)	*P* = 0.02

In Datasets 1, 2 and 3, a light gradient boosting machine performed best. In Dataset 4, a multinomial naïve Bayes model performed best. The rightmost column is the result of a Wilcoxon nonparametric test comparing the best machine learning model and the best polygenic risk scoring approach. AUC = area under the receiver operator characteristic curve; CI = confidence interval.

^a^Lead variants from risk loci identified in the 2022 genome-wide association study meta-analysis^10^ (108 variants).

^b^All variants with *P*-value <5 × 10^−8^ identified from the 2022 genome-wide association study meta-analysis^10^ summary statistics (7771 variants).

^c^All variants with a *P*-value <1 × 10^−5^ identified from the re-calculated summary statistics without 23andMe (7840 variants).

^d^All linkage disequilibrium independent variants among all genotyped variants (140 467 variants).

### Model explainability

**Figure 4:**
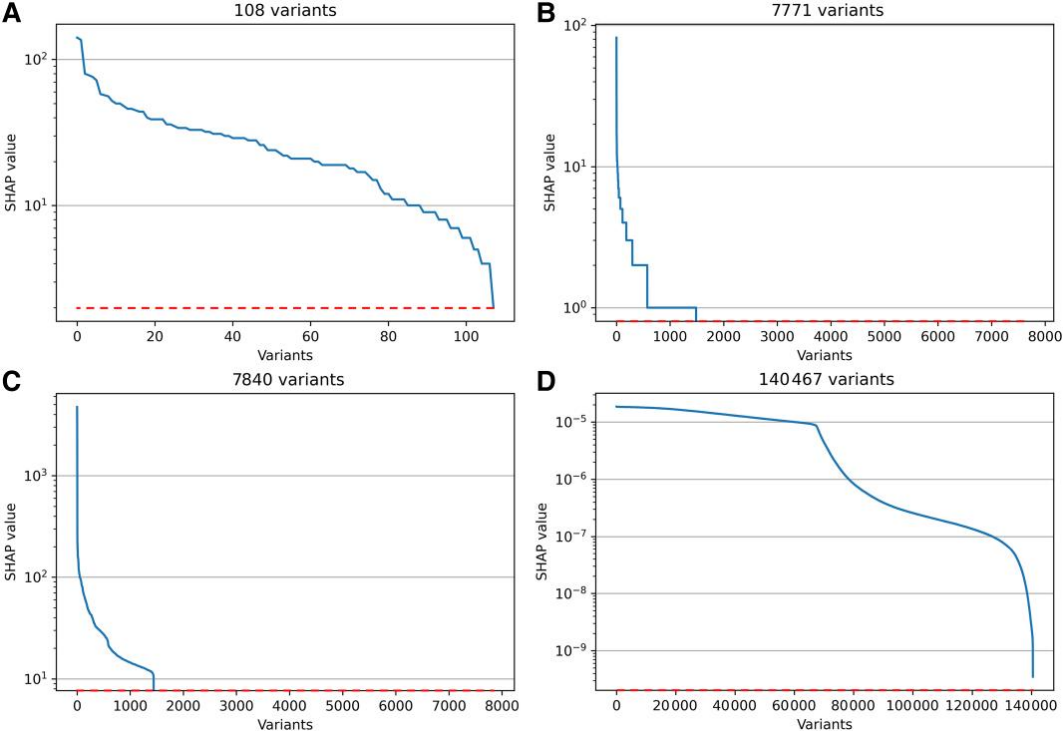
**SHAP summary plots.** Plots illustrating the relative contribution of the included variants to the predictions for the best machine learning model for each dataset. The *x*-axes denote number of variants, the *y*-axes denote the absolute SHAP value on a logarithmic scale. (**A**) In Dataset 1, all 108 variants contributed towards the prediction. (**B**) In Dataset 2, 1486 of 7771 variants contributed. (**C**) In Dataset 3, 1442 of 7840 variants contributed. In the two latter cases, a large majority of variants do not contribute to the prediction suggesting that the model omits the less important variants, however, still achieving higher accuracy than polygenic risk scoring suggesting that some non-additive effects between the contributing variants are captured. (**D**) In Dataset 4, all 140 467 variants contribute but with small contribution each. This is due to the probabilistic additive architecture of the naive Bayes approach, more similar to polygenic risk scoring. SHAP = Shapley additive explanations.

Figure 4 visualizes the SHAP values for the top performing machine learning models for each dataset. These figures demonstrate that Datasets 1 through 3 benefit from a model that may capture non-additive effects, whereas this advantage is lost in the largest dataset in favour of an additive probabilistic architecture. After ordering the variants by Shapley values, the top 123 variants were compared to those in the 2022 GWAS meta-analysis.^9^ As expected, all 108 variants in Dataset 1 were identified among the 123 from the GWAS meta-analysis. For the larger datasets, the number of common variants among the 123 most important were 13, 8 and 0 for Datasets 2, 3 and 4, respectively (Supplementary Table 4). Supplementary Fig. 2 shows the probability distribution plot and calibration plots for the top performing machine learning model and PRS. Cases and controls showed largely overlapping prediction probability density plots, however, more so for PRS as compared to the machine learning models.

### Gene annotation and pathway enrichment

**Figure 5:**
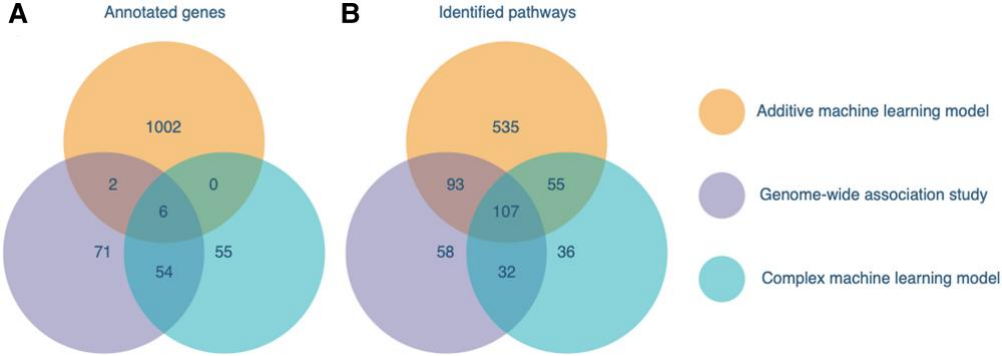
**Venn diagrams showing overlap of annotated genes and enriched pathways**. (**A**) Overlap of annotated genes from variants identified in the genome-wide association study, the best complex model (light gradient boosting machine in Dataset 2) and the best additive machine learning model (multinomial naïve Bayes in Dataset 4). (**B**) Overlap of enriched pathways from genes and variants identified in the genome-wide association study, the best complex model (light gradient boosting machine in Dataset 2) and the best additive machine learning model (multinomial naïve Bayes in Dataset 4).

All 108 variants for Dataset 1, the top 184 variants for Dataset 2, the top 123 variants for Dataset 3 and the top 1018 variants for Dataset 4 were used for gene annotations and pathway enrichment. In both Dataset 2 and Dataset 4, several variants were considered equally important; thus, these numbers exceeded 123 (184 and 1018, respectively). Table 3 and Fig. 5 detail the annotated genes and enriched pathways found to be common with those identified in the GWAS meta-analysis and those unique to the machine learning models.

**Table 3:** Comparison of identified genes and pathways

	Dataset 1 (108 variants)^a^	Dataset 2 (7771 variants)^b^	Dataset 3 (7840 variants)^c^	Dataset 4 (140 467 variants)^d^
Common genes, *n*/*N* (%)	73/105 (69.5)	60/115 (52.2)	38/95 (40.0)	8/1010 (0.8)
Genes unique to machine learning, *n*/*N* (%)	32/105 (30.5)	55/115 (47.8)	57/95 (60.0)	1002/1010 (99.2)
Common pathways, *n*/*N* (%)	228/254 (89.8)	139/230 (60.4)	127/226 (56.2)	200/790 (25.3)
Pathways unique to machine learning, *n*/*N* (%)	26/254 (10.2)	91/230 (39.6)	99/226 (43.8)	590/790 (74.7)

After gene annotation and pathway enrichment, the significant genes and pathways identified in the best machine learning models were compared to those identified in through a genome-wide association study approach.

^a^Lead variants from risk loci identified in the 2022 genome-wide association study meta-analysis^10^ (108 variants).

^b^All variants with *P*-value <5 × 10^−8^ identified from the 2022 genome-wide association study meta-analysis^10^ summary statistics (7771 variants).

^c^All variants with a *P*-value <1 × 10^−5^ identified from the re-calculated summary statistics without 23andMe (7840 variants).

^d^All linkage disequilibrium independent variants among all genotyped variants (140 467 variants).

Gene comparison analysis resulted in the identification of 55 genes that were unique to the best interactive machine learning model (light gradient boosting machine in Dataset 2), and 1002 genes that were unique to the best probabilistic/additive machine learning model (multinomial naive Bayes in Dataset 4). Furthermore, pathway enrichment analysis resulted in the identification of 91 and 590 additional pathways for the respective machine learning models. After pruning the identified pathways based on crude *P*-value threshold, 14 and 74 pathways were evaluated in detail (Supplementary Tables 5 and 6). Among these, 21 were considered significant after correcting for a false discovery rate of 0.1. The enriched pathways were primarily related to signal transduction and neurological function.

## Discussion

In this study, we found that machine learning outperforms PRS in distinguishing individuals with migraine from headache-free individuals based on genotype data. The best machine learning model achieved a hold-out test set AUC of 0.63, and the best PRS model achieved a hold-out test set AUC of 0.59. This is the first study to utilize machine learning to classify individuals with migraine and headache-free controls using genotype data.^38^ Other studies aiming to classify headaches have mainly focused on clinical data, MRI data or other non-genetic paraclinical data.^38-40^

Although an AUC of 0.63 is modest in absolute terms, it is the incremental performance of flexible models over PRS that is critical here. Gene-environment interactions and the imprecision of single point disease prevalence set a comparatively low ceiling on maximal achievable performance from genotypic data alone.^41^ But the substantial difference between twin study estimates of heritability and PRS performance^9,12^ suggests genetic susceptibility may be mediated by wider and more complex genetic interactions than conventional PRS models are able to capture, as evidenced by the superior performance of the flexible models used in our study. Note that the comparison between machine learning and PRS was stacked in favour of PRS here, since the PRS was based on a meta-analysis of 94 283 migraine cases and 738 834 controls, while the machine learning models were based on the much smaller HUNT study population. Hence, if compared between datasets of equal size, the magnitude of difference would be expected to be even larger in favour of machine learning.

Indeed, the findings from the three smallest datasets (108, 7771 and 7840 variants) support a non-additive and interactive genetic architecture for migraine, and can also explain why the machine learning approach outperforms PRS. Recall that the top performing model in these datasets was a light gradient boosting classifier, a model that can capture both non-linear relationships and interactions. Therefore, the observed superiority of these models over the purely additive PRS supports that the missing heritability may in part be attributed to non-additive effects such as gene-gene interactions. The notion that machine learning models can pick up non-linear and interactive effects of genotype is supported by empirical data from several other complex traits.^42^ In an analysis of 34 702 individuals from eight U.S. cohorts, an extreme gradient boosting model was demonstrated to increase the variance explained, compared to PRS, between 22% and 100% for complex traits such as height, blood pressure and cholesterol levels.^42^ That study supports our finding that complex machine learning models can capture non-linear and interactive effects also in migraine. It is further supported by several studies that have found that specific gene-gene interactions synergistically increase the susceptibility to migraine.^43-45^

We observe that the complex machine learning models show a small, but gradual, increase in performance with increasing genetic dimensionality before performance dramatically deteriorates beyond 93 237 variants (Fig. 3). On the other hand, the simpler probabilistic naïve Bayes models show a steady increase in performance before reaching a plateau beyond 57 965 variants. These patterns can be explained as follows.

The machine learning models perform only slightly better in Datasets 2 and 3 as compared to Dataset 1, likely because information from the same relatively small set of loci is used across all three models. This is evident from the SHAP plot (Fig. 5), where the light gradient boosting seems to prioritize slightly less than one fifth of variants. Notably, Dataset 3 used a higher *P*-value threshold for association but was drawn from a smaller sample, likely resulting in identifying variants from the same set of loci as Datasets 1 and 2.

When information from additional parts of the genome is incorporated in the models in the intermediate *post hoc* analyses (recall that these datasets used increasingly higher *r*^2^ cut-offs), it led to an increase in performance, before the sudden stall beyond 93 237 variants. This drop in performance can be explained by overfitting when feature dimensionality and complexity surpass what can be supported by the available sample size. A rule of thumb states that there should be at a minimum 5–10 samples for each feature (or dimension).^46^ However, despite performance initially increasing as the number of dimensions increases, beyond a certain dimensionality, the performance deteriorates.^46^

On the contrary, the relatively ‘simpler’ multinomial naïve Bayes model, assuming an additive probabilistic architecture, similar to PRS, plateaus beyond 57 965 variants where additional small-effect-variants provide negligible additional performance. This plateauing is, as expected, also observed for PRS (Fig. 3). In summary, we argue that complex models capture non-additive effects as long as the feature-to-sample ratio is appropriate, beyond which the simpler models are favoured. This paradoxical phenomenon supports the second explanation for the ‘missing heritability’, namely that there are many small-to-medium sized variants that fail to reach the genome-wide significance threshold but have an impact on PRS and additive models such as naïve Bayes.

In this study, we identified several genes and pathways that seem to be unique to the machine learning approach. While the best model for the smallest dataset primarily identified genes and pathways already established in the GWAS meta-analysis, the complex models of Datasets 2 and 3 resulted in several unique genes and pathways. The majority of the most important variants, as identified by the Shapley analysis, were also unique for Datasets 2 and 3. The genes annotated to these variants were primarily enriched in pathways related to signal transduction and neurological function, which is biologically plausible for migraine. Dataset 4 with 140 167 variants and a probabilistic naïve Bayes model resulted in almost exclusively unique genes. This is likely due to the larger number of variants included in the annotation and pathway analysis, naturally leading to inclusion of a wider part of the genome and thus significantly more genes and pathways. Therefore, any biological interpretations from this dataset must be done with caution.

Interestingly, the overall best model (light gradient boosting machine in Dataset 2) highlighted pathways related to calcitonin gene-like receptors and the toxicity of botulinum toxin A, D, E and F. The calcitonin gene-related ligand and its receptor play an important role in migraine pathophysiology, where they mediate trigeminovascular pain transmission and vasodilatory neurogenic inflammation.^47^ They are also targets of several monoclonal antibodies that have demonstrated effectiveness in preventing migraine.^48^ One of the risk loci identified in the 2022 GWAS meta-analysis contains the gene encoding calcitonin gene-related peptide itself, but not its receptor.^9^ The identification of the receptor in this study suggests that both the ligand and receptor are relevant for the susceptibility of migraine. OnabotulinumtoxinA is a therapeutic agent used as preventive treatment for migraine.^49^ Its mechanism of action is thought to be the inhibition of pro-inflammatory and excitatory neurotransmitters and neuropeptides from primary afferent nociceptive pain fibres in the head and neck that participate in the development of peripheral and central sensitization.^50^ The pathway identified here involves the SV2A gene, encoding synaptic vesicle glycoprotein 2A, which has been shown to be the receptor for botulinum toxin A.^51^ It is therefore conceivable that an upregulation of the receptor increases the susceptibility to both migraine and the treatment effect of OnabotulinumtoxinA.

The approach of complex genotype modelling has several potential downstream clinical implications. First, future models with improved performance could serve as an objective measure of migraine. Second, the modelling approach is transferrable and could prove a valuable risk scoring tool for other phenotypically diverse, idiopathic neurological traits of non-additive genetic architecture. Finally, further unravelling of the model architecture could help elucidate the underlying aetiology and pathophysiology of migraine, paving the way for clinical and therapeutic markers.

We believe that complex models that can capture both interactive and additive effects will further improve classification by genotype, given a sufficiently large sample. The prerequisites for such models to be successful rely on sufficiently large sample sizes to allow complex modelling without overfitting and the use of the right computational algorithms, such as non-linear machine learning models and deep neural networks. Future efforts to classify migraine by genotypic data adhering to these prerequisites are likely to outperform the classification performance of this study. Moreover, future studies should aim to incorporate demographic, phenotypic and other medical data that could further take advantage of important gene-environment and epigenetic factors that most likely partake in migraine aetiology.^12^ Finally, it is important that future research efforts also aim to validate the models in out-of-sample cohorts to assess their generalizability.

### Strengths and limitations

This paper has several strengths. First, the models are rigorously validated in a held-out unseen test set. The test set performances are faithful to the trained model, suggesting that it is generalizable. Secondly, the developed models are compared to a validated standard, namely PRS, which establishes their robustness. Both strengths overcome challenges that are repeatedly cited as barriers to why machine learning fails to prove clinically useful in medicine.^38,52^ Thirdly, the models are free from any apparent data leaks, contrary to what typically happens when the input for the classification models is the symptomatology of the migraine, which is what determines the headache status, thus leading to overly optimistic classification results. Weaknesses of the study include the moderate sensitivity of the phenotype assignment, although with near-perfect specificity—a potential classification bias. However, because migraine is the minority class, with lower sensitivity, the class imbalance increases, thereby creating a more challenging classification task, which ultimately leads to underestimation of the model precision. Another limitation is that we were not able to remove the influence of a few HUNT individuals in the calculation of summary statistics, which could have biased the models in favour of the dataset at hand. Nevertheless, this limitation is expected to increase the performance of the PRS; hence, this weakness does not invalidate the finding that machine learning outperforms PRS.

When comparing machine learning and PRS, there are several strengths and weaknesses of both approaches that should be acknowledged. The most important strength of the machine learning models for the task at hand is the ability to capture non-additive and interactive effects. However, it comes at the cost of often high computational time and limited interpretability. PRS, on the other hand, is a validated and commonly accepted method of assessing the risk of complex traits and is much less computationally expensive.^30^ Still, it is limited to assessing additive genetic architectures, which is likely insufficient for migraine.^12^

## Conclusion

In this study, we demonstrate that machine learning outperforms PRS in distinguishing migraine from headache-free controls when using genome-wide genotype data and succeed in identifying new genes and pathways potentially implicated in the disease. Complex machine learning models significantly outperform PRS when the number of genetic variants is relatively low, supporting a non-additive and interactive genetic architecture. However, this benefit diminishes with increasing input dimensionality in favour of additive effects. Our findings support both an additive and an interactive and non-additive genetic basis for migraine, validating the hypothesized explanations for the missing heritability. Future research investigating larger cohorts with complex models that capture both additive and interactive relationships could likely improve classification performance based on genotype.

## Supplementary Material

awaf172_Supplementary_Data

## Data Availability

The minimum dataset required to replicate this work contains personal sensitive information and is not publicly available nor available upon request. The analytical code may be provided upon reasonable request.

## Appendix 1

International Headache Genetics Consortium

Full details are provided in the Supplementary material.

Verneri Anttila, Ville Artto, Andrea C. Belin, Anna Bjornsdottir, Gyda Bjornsdottir, Dorret I. Boomsma, Sigrid Børte, Mona A. Chalmer, Daniel I. Chasman, Bru Cormand, Ester Cuenca-Leon, George Davey-Smith, Irene de Boer, Martin Dichgans, Tonu Esko, Tobias Freilinger, Padhraig Gormley, Lyn R. Griffiths, Eija Hämäläinen, Thomas F. Hansen, Aster V. E. Harder, Heidi Hautakangas, Marjo Hiekkala, Maria G. Hrafnsdottir, M. Arfan Ikram, Marjo-Riitta Järvelin, Risto Kajanne, Mikko Kallela, Jaakko Kaprio, Mari Kaunisto, Lisette J. A. Kogelman, Espen S. Kristoffersen, Christian Kubisch, Mitja Kurki, Tobias Kurth, Lenore Launer, Terho Lehtimäki, Davor Lessel, Lannie Ligthart, Sigurdur H. Magnusson, Rainer Malik, Bertram Müller-Myhsok, Carrie Northover, Dale R. Nyholt, Jes Olesen, Aarno Palotie, Priit Palta, Linda M Pedersen, Nancy Pedersen, Matti Pirinen, Danielle Posthuma, Patricia Pozo-Rosich, Alice Pressman, Olli Raitakari, Caroline Ran, Gudrun R. Sigurdardottir, Hreinn Stefansson, Kari Stefansson, Olafur A. Sveinsson, Gisela M. Terwindt, Thorgeir E. Thorgeirsson, Arn M. J. M. van den Maagdenberg, Cornelia van Duijn, Maija Wessman, Bendik S. Winsvold, John-Anker Zwart.
