# Supplementary material for: Diagnosing migraine from genome-wide genotype data: a machine learning analysis
Source: Brain. 2025 May 6;149(1):290–301. doi: 10.1093/brain/awaf172 (PMC12782171; doi:10.1093/brain/awaf172)
Supplement: awaf172_Supplementary_Data [file awaf172_supplementary_data.zip › brain-2024-03043-File010.pdf]

International Headache Genetics Consortium

Verneri Anttila[1,2,3], Ville Artto[4], Andrea C Belin[5], Anna Bjornsdottir[6], Gyda Bjornsdottir[7], Dorret I Boomsma[8], Sigrid Børte[9,10,11], Mona A Chalmer[12], Daniel I Chasman[13,14], Bru Cormand[15], Ester Cuenca-Leon[16], George Davey-Smith[17], Irene de Boer[18], Martin Dichgans[19,20], Tonu Esko[21], Tobias Freilinger[22,23], Padhraig Gormley[24], Lyn R Griffiths[25], Eija Hämäläinen[26], Thomas F Hansen[12,27], Aster VE Harder[18,28], Heidi Hautakangas[26], Marjo Hiekkala[29], Maria G Hrafnsdottir[30], M. Arfan Ikram[31], Marjo-Riitta Järvelin[32,33,34,35], Risto Kajanne[26], Mikko Kallela[4], Jaakko Kaprio[26], Mari Kaunisto[29], Lisette JA Kogelman[12], Espen S Kristoffersen[36,37,38], Christian Kubisch[39], Mitja Kurki[40], Tobias Kurth[41], Lenore Launer[42], Terho Lehtimäki[43], Davor Lessel[39], Lannie Ligthart[8], Sigurdur H Magnusson[7], Rainer Malik[19], Bertram Müller-Myhsok[44], Carrie Northover[45], Dale R Nyholt[46], Jes Olesen[12], Aarno Palotie[26,47], Priit Palta[26], Linda M Pedersen[48], Nancy Pedersen[49], Matti Pirinen[26,50,51], Danielle Posthuma[52], Patricia Pozo-Rosich[53], Alice Pressman[54], Olli Raitakari[55,56,57], Caroline Ran[5], Gudrun R Sigurdardottir[6], Hreinn Stefansson[7], Kari Stefansson[7], Olafur A Sveinsson[30], Gisela M Terwindt[18], Thorgeir E Thorgeirsson[7], Arn MJM van den Maagdenberg[18,28], Cornelia van Duijn[58], Maija Wessman[29,26], Bendik S Winsvold[48,9,59], John-Anker Zwart[48,9,10]

[1]Analytical and Translational Genetics Unit, Department of Medicine, Massachusetts General Hospital and Harvard Medical School, Boston, Massachusetts, USA, [2]Program in Medical and Population Genetics, Broad Institute of MIT and Harvard, Cambridge, Massachusetts, USA, [3]Stanley Center for Psychiatric Research, Broad Institute of MIT and Harvard, Cambridge, Massachusetts, USA, [4]Department of Neurology, Helsinki University Central Hospital, Helsinki, Finland, [5]Department of Neuroscience, Karolinska Institutet, Stockholm, Sweden, [6]Neurology private practice, Laeknasetrid, Reykjavik, Iceland, [7]deCODE genetics/Amgen Inc., Reykjavik, Iceland, [8]Netherlands Twin Register, Department of Biological Psychology, Vrije Universiteit, Amsterdam, the Netherlands, [9]K.G. Jebsen Center for Genetic Epidemiology, Department of Public Health and Nursing, Faculty of Medicine and Health Sciences, Norwegian University of Science and Technology, Trondheim, Norway, [10]Institute of Clinical Medicine, Faculty of Medicine, University of Oslo, Oslo, Norway, [11]Research and Communication Unit for Musculoskeletal Health, Department of Research, Innovation and Education, Division of Clinical Neuroscience, Oslo University Hospital, Oslo, Norway, [12]Danish Headache Center, Department of Neurology, Copenhagen University Hospital,

Copenhagen, Denmark, [13]Department of Medicine, Division of Preventive Medicine, Brigham and Women's Hospital, Boston, Massachusetts, USA, [14]Harvard Medical School, Boston, Massachusetts, USA, [15]Department of Genetics, Spain Centre for Biomedical Network Research on Rare Diseases, University of Barcelona, Barcelona, Spain, [16]Pediatric Neurology Research Group, Vall d'Hebron Research Institute, Barcelona, Spain, [17]University of Bristol/Medical Research Council Integrative Epidemiology Unit, University of Bristol, Bristol, UK, [18]Department of Neurology, Leiden University Medical Centre, Leiden, the Netherlands, [19]Institute for Stroke and Dementia Research, University Hospital, LMU Munich, Munich, Germany, [20]Munich Cluster for Systems Neurology, Munich, Germany, [21]Estonian Biobank Registry, the Estonian Genome Center, University of Tartu, Tartu, Estonia, [22]Department of Neurology, Klinikum Passau, Passau, Germany, [23]Department of Neurology and Epileptology, Hertie Institute for Clinical Brain Research, University of Tuebingen, Tuebingen, Germany, [24]GSK Inc., Cambridge, Massachusetts, USA, [25]Centre for Genomics and Personalised Health, Queensland University of Technology, Brisbane, Queensland, Australia, [26]Institute for Molecular Medicine Finland, Helsinki Institute of Life Science, University of Helsinki, Helsinki, Finland, [27]Novo Nordic Foundation Center for Protein Research, Copenhagen University, Copenhagen, Denmark, [28]Department of Human Genetics, Leiden University Medical Centre, Leiden, the Netherlands, [29]Folkhälsan Research Center, Helsinki, Finland, [30]Landspitali University Hospital, Reykjavik, Iceland, [31]Department of Epidemiology, Erasmus University Medical Center, Rotterdam, the Netherlands, [32]Department of Epidemiology and Biostatistics, MRC-PHE Centre for Environment and Health, School of Public Health, Imperial College London, London, UK, [33]Center for Life Course Health Research, Faculty of Medicine, University of Oulu, Oulu, Finland, [34]Unit of Primary Health Care, Oulu University Hospital, OYS, Oulu, Finland, [35]Department of Life Sciences, College of Health and Life Sciences, Brunel University London, London, UK, [36]Research and Communication Unit for Musculoskeletal Health, Department of Research, Innovation and Education, Division of Clinical Neuroscience, Akershus University Hospital and University of Oslo, Oslo, Norway, [37]Department of General Practice, Institute of Health and Society, University of Oslo, Oslo, Norway, [38]Department of Neurology, Akershus University Hospital, Lørenskog, Norway, [39]Institute of Human Genetics, University Medical Center Hamburg-Eppendorf, Hamburg, Germany, [40]Psychiatric and Neurodevelopmental Genetics Unit, Department of Medicine, Massachusetts General Hospital, Boston, Massachusetts, USA, [41]Institute of Public Health, Charité – Universitätsmedizin, Berlin, [42]Laboratory of Epidemiology and Population Sciences, Intramural Research Program, National Institute on

Aging, Bethesda, Maryland, USA, [43]Department of Clinical Chemistry, Fimlab Laboratories, and Finnish Cardiovascular Research Center - Tampere, Faculty of Medicine and Health Technology, Tampere University, Tampere, Finland, [44]Max Planck Institute of Psychiatry, Munich, Germany, [45]23&Me Inc., Mountain View, California, USA, [46]School of Biomedical Sciences, Faculty of Health, Centre for Genomics and Personalised Health, Centre for Data Science, Queensland University of Technology, Brisbane, Queensland, Australia, [47]University of Helsinki, Helsinki, Finland, [48]Department of Research, Innovation and Education, Division of Clinical Neuroscience, Oslo University Hospital, Oslo, Norway, [49]Department of Medical Epidemiology and Biostatistics, Karolinska Institutet, Stockholm, Sweden, [50]Department of Mathematics and Statistics, University of Helsinki, Helsinki, Finland, [51]Department of Public Health, University of Helsinki, Helsinki, Finland, [52]Department of Complex Trait Genetics, Center for Neurogenomics and Cognitive Research, Neuroscience Campus Amsterdam, VU University, Amsterdam, The Netherlands, [53]Headache Unit, Neurology Department, Vall d'Hebron University Hospital, Barcelona, Spain, [54]Sutter Health, Sacramento, California, USA, [55]Centre for Population Health Research, University of Turku, Turku University Hospital, Turku, Finland, [56]Research Centre of Applied and Preventive Cardiovascular Medicine, University of Turku, Turku, Finland, [57]Department of Clinical Physiology and Nuclear Medicine, Turku University Hospital, Turku, Finland, [58]Department of Epidemiology, Erasmus University Medical Centre, Rotterdam, the Netherlands, [59]Department of Neurology, Oslo University Hospital, Oslo, Norway
