# Supplementary material for: Diagnosing migraine from genome-wide genotype data: a machine learning analysis
Source: Brain. 2025 May 6;149(1):290–301. doi: 10.1093/brain/awaf172 (PMC12782171; doi:10.1093/brain/awaf172)
Supplement: awaf172_Supplementary_Data [file awaf172_supplementary_data.zip › brain-2024-03043-File011.pdf]

SUPPLEMENTARY MATERIAL

Supplementary figure 1. Flow-chart of study population

Supplementary figure 2. Probability distribution plots and calibration plots of the top performing models

Supplementary table 1. Python packages.

Supplementary table 2. Sensitivity analysis of feature dimensionality.

Supplementary table 3. Relatedness sensitivity analysis.

Supplementary table 4. Most important variants ordered by Shapley values.

Supplementary table 5. 14 additional pathways identified by the best performing interactive machine learning model

Supplementary table 6. 74 additional pathways identified by the best performing probabilistic/additive machine learning model

Supplementary figure 1. Flow-chart of study population

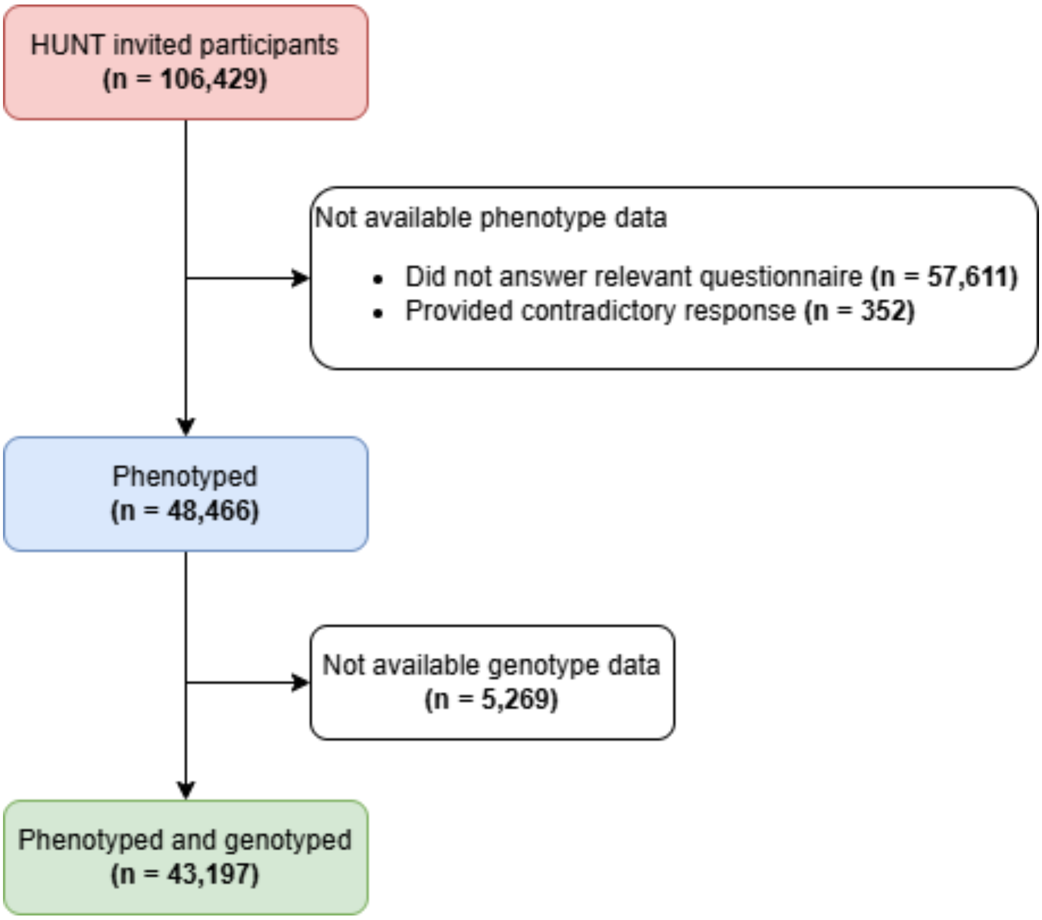

Supplementary Figure 2. Probability distribution plots and calibration plots of the top performing machine learning model (upper two plots) and the corresponding PRS model (lower two plots). The plots were constructed based on the models' predictions in the best performing test set (dataset 3; 7,840 variants). The models rarely predict high probabilities for neither cases nor controls, indicating the limited distinguishable information is being picked up from genetic data, however, the predicted probabilities are more discernable for machine learning. For each point on the calibration plot, the y-axis denotes the proportion of true outcomes, and the x-axis denotes the mean predicted probability. Therefore, a well-calibrated model has a calibration plot that converges to the straight dotted diagonal line. Both models converge away from perfect calibration further supporting the difficult separation of cases and controls.

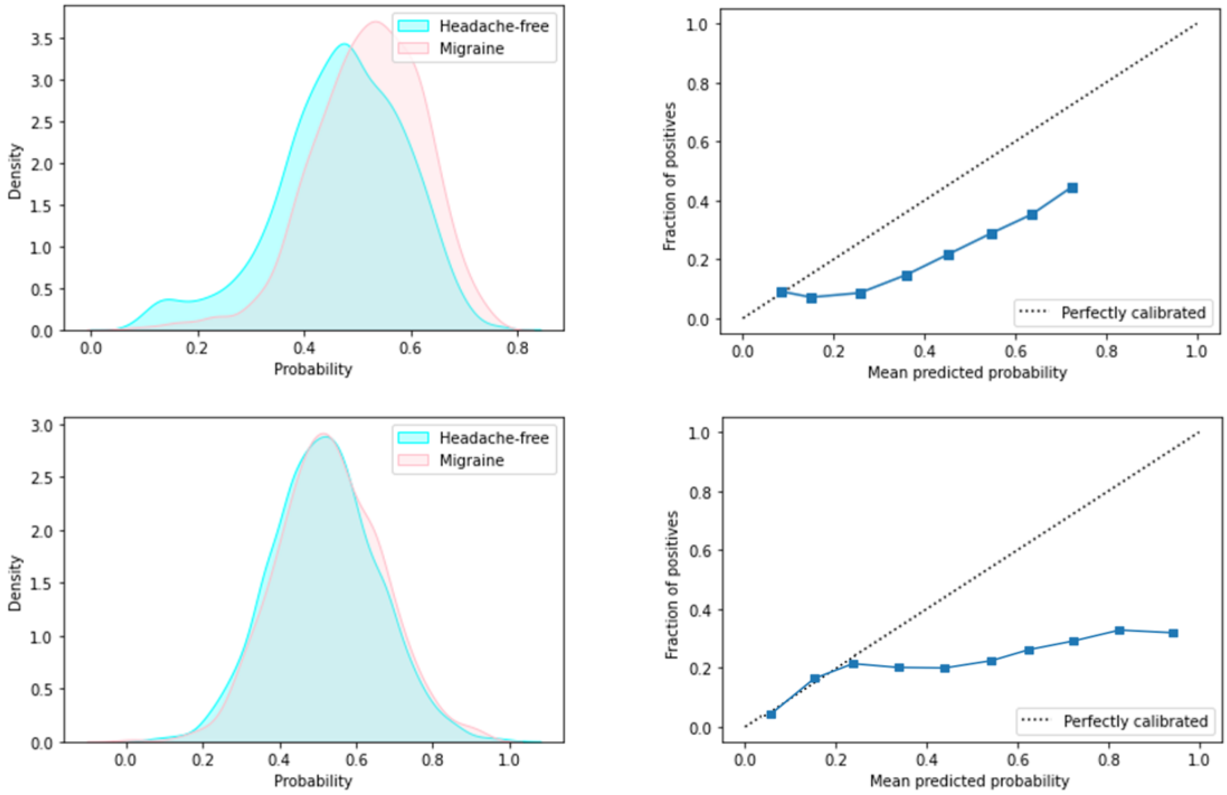

Supplementary table 1. Python packages.

Python v3.10	
**Package**	**Version**
bayesian-optimization	1.4.3
catboost	1.2.2
dask	2024.3.1
dask-ml	2023.3.24
FLAML	2.1.2
ForestDiffusion	1.0.5
estimators	0.1.0
networkx	3.4.2
imbalanced-learn	0.12.0
keras	2.12.0
lightgbm	4.3.0
matplotlib	3.8.2
numpy	1.23.1
openml	0.14.2
optuna	3.6.0
pandas	2.2.0
pgmpy	0.1.5
pysam	0.22.0
pytorch-tabnet	4.1.0
scikit-learn	1.2.1
scikit-optimize	0.10.1
scipy	1.12.0
seaborn	0.13.2
shap	0.46.0
tensorflow	2.12.0
vcfpy	0.13.8
xgboost	2.0.3

Supplementary table 2. Sensitivity analysis of feature dimensionality.

Variants	**140,467**	**114,179**	**93,237**	**71,188**	**57,965**	**19,473**	**7,840**	**7,771**	**108**
**Complex optimized light gradient boosting model**									
AUC score	0.51 (0.51)	0. 52 (0.53)	0.66 (0.66)	0.64 (0.64)	0.66 (0.66)	0.62 (0.62)	0.63 (0.65 +/- 0.012)	0.63 (0.64 +/- 0.010)	0.63 (0.64 +/- 0.010)
Accuracy	0.75 (0.74)	0.72 (0.71)	0.64 (0.65)	0.63 (0.62)	0.64 (0.64)	0.62 (0.62)	0.62 (0.61 +/- 0.009)	0.61 (0.62 +/- 0.008)	0.60 (0.60 +/- 0.009)
F-1 score	0.02 (0.02)	0. 14 (0.15)	0.44 (0.42)	0.43 (0.41)	0.59 (0.58)	0.56 (0.56)	0.57 (0.58 +/- 0.010)	0.55 (0.55 +/- 0.009)	0.55 (0.55 +/- 0.009)
Recall	0.99 (0.99)	0. 92 (0.91)	0.67 (0.67)	0.65 (0.64)	0.62 (0.62)	0.58 (0.57)	0.60 (0.60 +/- 0.011)	0.59 (0.60 +/- 0.009)	0.59 (0.59 +/- 0.010)
Precision	0.01 (0.01)	0. 10 (0.10)	0.57 (0.56)	0.57 (0.57)	0.62 (0.62)	0.58 (0.57)	0.60 (0.60 +/- 0.011)	0.59 (0.60 +/- 0.009)	0.59 (0.59 +/- 0.010)
**Simple MultinomialNB model**									
AUC score	0.62 (0.62)	0.62 (0.62)	0.62 (0.62)	0.61 (0.62)	0.61 (0.61)	0.60 (0.61)	0.58 (0.59)	0.58 (0.59)	0.58 (0.58)
Accuracy	0.58 (0.59)	0.61 (0.62)	0.64 (0.64)	0.67 (0.67)	0.70 (0.70)	0.71 (0.70)	0.55 (0.55)	0.55 (0.55)	0.56 (0.57)
F-1 score	0.43 (0.45)	0.47 (0.47)	0.52 (0.53)	0.55 (0.55)	0.54 (0.54)	0.55 (0.55)	0.51 (0.52)	0.51 (0.52)	0.51 (0.53)
Recall	0.56 (0.57)	0.56 (0.56)	0.56 (0.56)	0.56 (0.56)	0.56 (0.56)	0.55 (0.55)	0.55 (0.56)	0.55 (0.56)	0.55 (0.56)
Precision	0.64 (0.65)	0.61 (0.62)	0.58 (0.59)	0.55 (0.56)	0.54 (0.55)	0.55 (0.55)	0.55 (0.56)	0.55 (0.56)	0.56 (0.56)

Values presented are test set metrics and validation set metrics in parenthesis. 10-fold cross validation was used for the datasets with 108 to 7,840 variants for the light gradient boosting model, thus the mean of the validation folds with one standard deviation is reported here. For the light gradient boosting model, the datasets that contained more than 7,840 variants were validated using one-fold-cross-validation (training validation in parenthesis) due to time and resource-intensiveness of the procedures. For the same reason, the multinomial naïve bayes model was also validated using one-fold-cross-validation for all datasets.

Supplementary table 3. Relatedness sensitivity analysis.

**Dataset 1 (light gradient boosting)**	**Without heritability control**	**With heritability control**
Accuracy	0.60 (0.60)	0.62 (0.61)
F-1 score	0.55 (0.55)	0.57 (0.56)
Recall	0.59 (0.59)	0.60 (0.59)
Precision	0.59 (0.59)	0.60 (0.59)
AUC score	0.63 (0.64 +/- 0.01)	0.63 (0.63 +/- 0.02)
**Dataset 2 (light gradient boosting)**	**Without heritability control**	**With heritability control**
Accuracy	0.61 (0.62)	0.63 (0.62)
F-1 score	0.55 (0.57)	0.57 (0.56)
Recall	0.59 (0.60)	0.59 (0.59)
Precision	0.59 (0.60)	0.59 (0.59)
AUC score	0.63 (0.64 +/- 0.01)	0.62 (0.63 +/- 0.01)
**Dataset 3 (light gradient boosting)**	**Without heritability control**	**With heritability control**
Accuracy	0.62 (0.61)	0.63 (0.62)
F-1 score	0.57 (0.57)	0.57 (0.56)
Recall	0.60 (0.60)	0.59 (0.59)
Precision	0.60 (0.60)	0.59 (0.59)
AUC score	0.64 (0.65 +/- 0.01)	0.63 (0.63 +/- 0.01)
**Dataset 4 (multinomial naive Bayes)**	**Without heritability control**	**With heritability control**
Accuracy	0.58 (0.57)	0.60 (0.58)
F-1 score	0.43 (0.39)	0.44 (0.43)
Recall	0.56 (0.56)	0.59 (0.57)
Precision	0.64 (0.61)	0.60 (0.63)
AUC score	0.62 (0.62)	0.61 (0.62)

Values presented are test set metrics and validation set metrics in parenthesis.

Supplementary table 4. Most important variants ordered by Shapley values. Variants from datasets 2, 3 and 4 are ordered by Shapley values. Variants in the GWAS meta-analysis are ordered by odds ratio. Chr: Chromosome; Pos: Position; Ref: Reference allele; Alt: Alternate allele.

	Top 123 variants dataset 2: 7,771 variants				Top 123 variants dataset 3: 7,840 variants				Top 123 variants dataset 4: 140,467 variants				Top 123 variants from GWAS Meta-analysis			
**No**	**Chr**	**Pos**	**Ref**	**Alt**	**Chr**	**Pos**	**Ref**	**Alt**	**Chr**	**Pos**	**Ref**	**Alt**	**Chr**	**Pos**	**Ref**	**Alt**
1	23	34102712	A	C	23	141572805	G	A	14	82830219	C	T	14	76496477	G	T
2	23	34053843	C	A	23	109912504	T	C	10	28225259	T	C	3	154289946	A	C
3	23	40744987	T	C	23	41127618	C	T	9	82448552	T	C	2	203832867	C	T
4	3	154323860	T	C	23	109917734	A	C	1	171947018	T	G	14	94844947	T	C
5	23	40747301	A	C	17	80979096	G	A	9	82187750	A	G	1	3075597	G	A
6	14	93591673	G	A	14	93595591	C	A	20	2042324	G	A	12	57527283	T	C
7	6	39187886	G	A	10	8773143	C	T	11	67540553	A	T	1	115677183	C	T
8	17	60720058	G	T	17	7366619	C	T	10	47701570	A	G	2	234825093	T	C
9	1	115677946	A	C	17	38253212	T	C	2	202816188	A	T	7	40427617	T	C
10	17	60636826	A	G	2	146066283	A	G	5	100818350	C	A	6	97059666	G	A
11	23	40745032	G	A	11	3247976	A	G	11	68792679	C	G	10	100702737	G	A
12	17	7366619	C	T	17	78395361	T	C	2	200448798	G	A	2	176978383	C	A
13	10	134587640	A	G	2	234826661	G	C	15	93918298	C	T	6	12903957	A	G
14	9	71812798	T	C	2	7996507	A	G	1	62361565	T	C	6	31850308	C	A
15	5	74988369	G	A	12	57532982	T	C	8	39311677	T	C	1	156450873	T	C
16	11	3247976	A	G	17	38252660	A	G	10	45017207	C	T	6	121846038	T	G
17	6	39191384	C	T	2	199253215	C	G	12	124744414	C	G	12	4527322	C	T
18	16	87575332	A	G	6	12922074	A	G	12	124747361	C	T	20	19469817	C	G
19	17	78243211	C	T	17	78337893	T	C	2	200743912	G	A	19	19406126	A	G
20	12	57532982	T	C	6	121776326	G	C	13	108844946	C	G	21	35593827	G	A
21	1	156407051	C	T	6	52212250	C	T	1	172054042	A	T	7	73013901	C	T
22	23	40748031	G	T	12	57265952	T	C	12	122964708	T	C	9	119258583	C	T
23	6	39177971	C	G	9	126708730	T	C	2	204789002	G	C	11	10674044	G	C
24	2	146249507	A	G	5	172645766	T	C	3	197052121	C	T	3	80302512	T	C
25	5	172648002	A	G	3	173658825	T	C	2	207683378	A	G	10	96039597	G	C

26	23	34070900	G	A	6	96988696	T	A	19	4513143	C	T	6	39183470	T	C
27	17	7328821	C	A	17	60544541	G	A	1	175046826	G	A	14	93595591	A	C
28	1	3085186	T	C	9	29394949	T	C	13	108646477	T	C	13	110788441	A	G
29	12	4530236	C	T	1	3085186	T	C	2	206800766	A	G	17	7366619	T	C
30	1	149881900	G	A	1	115677946	A	C	7	56037945	G	A	1	174601659	G	C
31	11	3249552	T	C	12	57525756	A	G	9	81155989	T	C	5	122306398	T	C
32	12	4515015	A	C	1	156409458	G	A	3	191728386	C	T	5	149380493	A	G
33	6	12922074	A	G	12	57527283	C	T	14	32395943	T	C	1	73891226	I	D
34	10	96129098	A	G	5	74932099	G	T	8	68981646	T	C	9	140743200	T	A
35	8	64496159	T	C	9	71782556	G	A	11	64574200	G	A	11	3249984	T	C
36	19	13361279	T	C	12	4549255	G	A	22	37697763	A	G	10	134479675	D	I
37	1	115658776	A	C	20	19528667	C	A	8	101923809	C	T	3	30472786	A	G
38	19	13355633	C	T	9	71838985	G	T	10	50818762	C	T	20	10684159	G	C
39	11	66401373	G	T	17	53412915	A	G	1	172935603	G	C	1	150510660	C	T
40	10	125272745	T	C	1	245822211	G	A	12	122129023	C	T	11	46548094	A	G
41	1	206777615	A	G	17	60636826	A	G	1	62003185	C	A	9	71746838	T	C
42	12	57524108	C	T	12	57540751	C	G	2	200600151	G	A	16	87578039	G	A
43	17	47514127	A	C	1	150464603	A	G	4	5035374	G	A	5	145752008	T	C
44	6	12768218	T	C	17	47513711	C	T	1	22940753	G	A	4	35469918	G	C
45	1	115677183	C	T	6	39090524	T	C	11	74476417	C	A	12	41901277	C	T
46	1	115657613	T	C	12	57506350	A	G	18	27988551	T	A	9	109687403	G	A
47	23	40774711	A	G	1	156403681	C	G	6	56585796	C	T	8	27266287	A	T
48	1	3217066	A	G	10	96039597	C	G	2	189765949	T	A	20	31168439	T	C
49	12	4549255	G	A	17	78337058	C	G	5	91869104	G	T	3	88210464	A	G
50	6	39090524	T	C	6	96960232	T	C	10	33601478	A	T	2	145258445	C	T
51	9	119234953	A	C	3	124550364	T	C	5	91749252	C	A	16	75442143	T	G
52	14	93596315	T	C	12	111281108	T	G	2	189132087	T	C	5	121515195	G	A
53	1	115820598	C	T	14	100111409	G	C	19	1773437	T	C	12	90091782	C	G
54	9	14103618	C	A	10	96792202	C	T	19	1764143	T	A	17	47514039	G	T

55	14	27698274	A	G	1	115676278	T	C	5	91556096	C	T	12	124820705	G	A
56	14	27699322	C	T	14	93591673	G	A	4	14200455	G	A	1	15538493	C	T
57	16	75331044	G	T	5	74988369	G	A	10	33252299	A	G	10	8722944	T	G
58	6	12911965	G	A	3	154323860	T	C	5	91029327	G	A	17	78256432	C	T
59	15	81025755	G	A	14	93596315	T	C	10	32750635	G	A	11	102070976	T	A
60	19	13350813	G	A	6	111658220	T	G	20	4851901	A	G	14	27661650	A	G
61	11	102105673	A	G	1	115658776	A	C	11	78772032	T	G	5	74963277	G	C
62	12	57506350	A	G	1	7046925	C	G	5	88773965	G	T	18	55192245	A	G
63	6	121776326	G	C	12	4515015	A	C	6	162596385	A	G	4	57727311	T	C
64	9	140743200	A	T	11	3251845	C	G	4	12377477	G	C	18	20201527	A	T
65	10	96039597	C	G	5	74934009	A	T	2	199766035	G	T	18	44866736	C	T
66	1	15558008	C	T	17	60757983	G	C	4	11242706	C	G	1	38366907	C	T
67	2	97051981	T	C	7	40484576	G	A	8	69775828	G	A	10	124230750	T	G
68	1	156464545	G	C	12	4549019	C	T	5	97577533	T	A	5	176676461	A	T
69	1	150257714	G	A	12	4527322	C	T	10	42607765	A	C	16	4534482	G	A
70	1	38373941	T	C	12	57524108	C	T	13	75207996	A	G	17	60720058	G	T
71	1	38371691	C	A	14	100121772	A	C	17	13376980	C	T	1	245847455	G	A
72	21	35596842	C	T	22	41418229	G	T	10	38675824	G	A	19	41864509	A	T
73	12	57540751	C	G	5	122264736	T	G	11	71381093	C	A	5	81129663	T	C
74	14	93582717	C	G	2	234762946	G	C	2	193024627	A	C	2	146037564	C	T
75	5	172619063	A	G	6	12826479	C	T	7	52763452	C	T	1	206843108	C	T
76	1	150399105	T	A	12	124970087	A	G	1	168465557	A	G	10	125242283	C	T
77	4	35549464	T	C	9	84642933	C	T	8	502511	A	G	11	30547438	C	T
78	20	19524558	T	C	2	7996361	G	A	6	54496854	T	A	9	29372501	C	G
79	2	204263036	G	A	4	20116802	G	A	1	168262624	A	G	10	104741114	C	A
80	14	27673481	C	T	6	121758512	T	C	5	95207168	G	T	2	96576609	A	T
81	14	93595591	C	A	21	35593827	A	G	4	8789929	T	C	15	81022364	A	G
82	12	57510661	C	T	17	47256685	D	I	11	74072965	G	C	6	30749712	T	G
83	1	73501065	D	I	21	28329468	G	A	4	11232637	C	T	8	64496159	T	C

84	11	10664033	T	G	1	3065568	T	C	8	9374455	A	C	7	120481569	C	T
85	9	71745663	A	G	7	71142219	T	C	5	106777494	G	T	6	22131929	C	A
86	12	57527283	C	T	6	12768218	T	C	13	76345298	T	A	1	39590409	T	G
87	6	22107290	G	A	9	86459262	D	I	1	180239636	C	T	6	111713302	A	G
88	11	46966633	C	T	17	60535836	A	G	1	180124916	C	T	20	45841052	G	A
89	12	4536134	A	G	17	47495796	C	T	8	66805131	T	A	11	15126085	A	G
90	10	125273539	A	G	9	136971029	T	C	10	60797143	C	A	13	47193696	G	A
91	10	125274226	A	G	17	1995254	G	A	2	219796436	C	T	22	20142932	C	T
92	10	134439478	C	T	3	30508604	T	C	1	236636954	G	A	10	21822856	G	A
93	10	134479675	D	I	6	96846764	A	T	8	66845510	A	G	17	1967501	C	T
94	1	156406381	G	C	9	119254725	C	T	17	6022961	C	T	6	72321017	T	C
95	17	47514039	G	T	6	22114325	T	C	11	45016652	G	C	5	172645766	T	C
96	19	41862253	G	A	6	22151747	C	T	2	218204845	G	A	14	58761912	G	A
97	18	20290616	T	C	2	234748973	G	A	2	217884460	G	C	2	171234235	G	C
98	20	31189993	C	T	10	105053558	A	C	14	33385120	C	T	19	13339128	G	A
99	12	57540004	D	I	2	7990214	C	T	1	59627098	A	G	6	150133954	C	A
100	6	121781390	T	C	19	13345088	T	C	15	91479286	C	T	12	98498223	A	C
101	6	121805199	C	T	14	69203677	A	G	9	4092292	C	T	1	92177663	G	T
102	20	19528667	C	A	2	211312565	T	C	22	44778329	G	A	2	43649780	A	C
103	5	121504647	C	G	12	57530670	C	T	2	217069101	T	C	3	86149109	T	A
104	12	4514858	T	C	18	20290616	T	C	17	12898994	C	G	3	124607055	C	T
105	10	100970599	C	T	11	7558570	C	T	22	45367385	T	C	21	36935896	C	T
106	23	40767514	A	C	2	112668189	G	A	15	90176251	G	A	1	60529980	C	A
107	6	96960016	C	T	12	57540004	D	I	2	216820354	C	T	1	66178918	G	A
108	23	40771535	G	C	1	115677183	C	T	15	34645422	A	G	17	46632679	G	C
109	7	72854549	T	C	1	15524381	G	A	11	41892576	A	T	14	42548912	G	A
110	23	40756587	C	G	10	71672879	T	C	16	78858473	C	T	14	75362552	T	C
111	23	34068582	C	T	2	204263036	G	A	2	224059154	G	A	1	186913055	G	T
112	16	87578626	G	A	1	3098360	G	A	15	35115310	G	A	X	40746484	T	C

113	11	46935185	C	G	6	22107290	G	A	9	5441950	G	A	11	61697078	A	T
114	19	13356150	T	G	2	146034665	G	A	6	165517355	C	T	11	66401373	G	T
115	16	75483064	T	G	2	145270592	A	G	14	33629693	G	A	17	77925681	C	T
116	23	40769058	G	A	1	3112417	C	T	8	92864072	T	C	9	14103618	A	C
117	2	43829540	T	C	10	124202675	T	C	5	117303076	T	C	1	7055843	T	C
118	6	97039857	G	A	21	35596842	C	T	2	223591125	A	G	11	133745852	G	A
119	10	8773143	C	T	5	122263970	T	C	12	76523945	A	G	10	112502662	A	C
120	1	3098644	A	G	6	96936682	G	A	1	235600590	G	A	13	78876537	T	C
121	1	38386727	A	G	3	30470027	T	C	10	62070777	T	C	2	156416638	G	A
122	7	40477363	A	G	1	115676054	D	I	12	117841986	G	A	3	48498456	T	A
123	1	73708637	G	A	14	94813402	G	A	5	116951547	A	G	X	34102712	A	C

Supplementary table 5. 14 additional pathways identified by the best performing interactive machine learning model.

Pathway Description	Parents	p-value	Adjusted FDR value	Genes
STAT6-mediated induction of chemokines	Immune System	0.009583	0.216058	STAT6
Toxicity of botulinum toxin type E (BoNT/E)	Disease	0.009583	0.216058	SV2A
**Toxicity of botulinum toxin type A (BoNT/A)**	**Disease**	**0.012757**	**0.216058**	**SV2A**
Toxicity of botulinum toxin type D (BoNT/D)	Disease	0.015922	0.216058	SV2A
Toxicity of botulinum toxin type F (BoNT/F)	Disease	0.015922	0.216058	SV2A
Defective CHST6 causes MCDC1	Disease	0.025356	0.216058	CHST6
Neurotoxicity of clostridium toxins	Disease	0.031597	0.216058	SV2A
**Calcitonin-like ligand receptors**	**Signal Transduction**	**0.031597**	**0.216058**	**CALCB**
Signaling by activated point mutants of FGFR1	Disease	0.034703	0.216058	FGF6
FGFR1c ligand binding and activation	Signal Transduction	0.037799	0.216058	FGF6
Mitochondrial iron-sulfur cluster biogenesis	Metabolism	0.040886	0.216058	FXN
FGFR2c ligand binding and activation	Signal Transduction	0.040886	0.216058	FGF6
FGFR4 ligand binding and activation	Signal Transduction	0.043963	0.216058	FGF6
Phospholipase C-mediated cascade; FGFR4	Signal Transduction	0.047030	0.216058	FGF6

The 14 additional pathways identified by the best performing interactive machine learning model (light gradient boosting machine in dataset 2), ranked by p-value. The second rightmost columns shows the p-value corrected for a false discovery rate of 0.1 using the Benjamini-Hochberg method. Biologically relevant genes from analyzed dataset are reported for each pathway. FDR: false discovery rate.

Supplementary table 6. 74 additional pathways identified by the best performing interactive machine learning model.

Pathway Description	Parents	p-value	Adjusted FDR value	Genes
ERBB2 Activates PTK6 Signaling	Signal Transduction	5.e-06	0.001770	ERBB3,ERBB4,NRG1, NRG2,NRG3
SHC1 events in ERBB2 signaling	Signal Transduction	6.e-06	0.001770	ERBB3,ERBB4,NRG1, NRG2,NRG3,PRKC
ERBB2 Regulates Cell Motility	Signal Transduction	1.2e-05	0.002360	ERBB3,ERBB4,NRG1, NRG2,NRG3
PI3K events in ERBB2 signaling	Signal Transduction	1.8e-05	0.002655	ERBB3,ERBB4,NRG1, NRG2,NRG3
PI3K events in ERBB4 signaling	Signal Transduction	4.3e-05	0.005074	ERBB4,NRG1,NRG2,N RG3
Long-term potentiation	Neuronal System	9.5e-05	0.007449	DLG2,ERBB4,GRIN2B, LRRC7,NRG1
Signaling by ERBB2 TMD/JMD mutants	Disease	9.5e-05	0.007449	ERBB3,ERBB4,NRG1, NRG2,NRG3
GRB7 events in ERBB2 signaling	Signal Transduction	0.000101	0.007449	ERBB3,NRG1,NRG2
Signaling by ERBB2 KD Mutants	Disease	0.000181	0.011269	ERBB3,ERBB4,NRG1, NRG2,NRG3
SHC1 events in ERBB4 signaling	Signal Transduction	0.000191	0.011269	ERBB4,NRG1,NRG2,N RG3
Signaling by ERBB2 in Cancer	Disease	0.000221	0.011854	ERBB3,ERBB4,NRG1, NRG2,NRG3
GRB2 events in ERBB2 signaling	Signal Transduction	0.000335	0.016471	ERBB4,NRG1,NRG2,N RG3
Downregulation of ERBB2 signaling	Signal Transduction	0.000377	0.017110	ERBB3,ERBB4,NRG1, NRG2,NRG3
Signaling by ERBB2	Signal Transduction	0.000744	0.031354	ERBB3,ERBB4,NRG1, NRG2,NRG3,PRKCE
Signaling by PTK6	Signal Transduction	0.001125	0.041484	ELMO1,ERBB3,ERBB4, NRG1,NRG2,NRG3
Signaling by Non-Receptor Tyrosine Kinases	Signal Transduction	0.001125	0.041484	ELMO1,ERBB3,ERBB4, NRG1,NRG2,NRG3
Constitutive Signaling by Aberrant PI3K in Cancer	Disease	0.001256	0.043591	ERBB3,ERBB4,ESR1,FG FR2,NRG1,NRG2,NR G3
Defective Mismatch Repair Associated with MSH2	Disease	0.001420	0.046544	MSH3,MSH6
Effects of PIP2 hydrolysis	Signal Transduction; Hemostasis	0.002316	0.071918	DGKB,DGKG,ITPR2,P RKCE
Downregulation of ERBB2:ERBB3 signaling	Signal Transduction	0.002540	0.074930	ERBB3,NRG1,NRG2
Repression of WNT target genes	Signal Transduction	0.003181	0.089371	LEF1,TCF7L2,TLE4
Cell junction organization	Cell-Cell communication	0.004051	0.108466	CDH13,CDH4,CTNN D1,DST,INADL,ITGB1 ,PARD6B
Diseases of Mismatch Repair (MMR)	Disease	0.004596	0.108466	MSH3,MSH6
MET interacts with TNS proteins	Signal Transduction	0.004596	0.108466	ITGB1,TNS3
MECP2 regulates transcription factors	Gene expression (Transcription)	0.004596	0.108466	PPARG,RBFOX1
Acetylcholine Neurotransmitter Release Cycle	Neuronal System	0.005660	0.128438	CHAT,RIMS1,SLC18A 3
Axon guidance	Developmental Biology	0.006243	0.136421	ANK3,ARHGEF11,AR HGEF28,CACNA1G,C NTNAP1,COL4A2,DC C,DNM3,DOK5,DOK 6,EFNA5,GRIN2B,ITG B1,KCNQ3,LHX4,NR P1,RELN,ROBO2,RPS9 ,SDC2,SRGAP3,VAV3
PI3K/AKT Signaling in Cancer	Disease	0.007107	0.143084	ERBB3,ERBB4,ESR1,FG FR2,NRG1,NRG2,NR G3
PI5P, PP2A and IER3 Regulate PI3K/AKT Signaling	Signal Transduction	0.007107	0.143084	ERBB3,ERBB4,ESR1,FG FR2,NRG1,NRG2,NR G3
Potassium Channels	Neuronal System	0.007487	0.143084	HCN2,KCNA2,KCNJ5 ,KCNMA1,KCNQ1,K CNQ3,KCNS3

Neurexins and neuroligins	Neuronal System	0.007518	0.143084	DLG2,EPB41L2,GRIN2B,NLGN1,NRXN3
Ras activation upon Ca2+ influx through NMDA receptor	Neuronal System	0.007809	0.143978	DLG2,GRIN2B,LRRC7
Unblocking of NMDA receptors, glutamate binding and activation	Neuronal System	0.009041	0.156888	DLG2,GRIN2B,LRRC7
Negative regulation of NMDA receptor-mediated neuronal transmission	Neuronal System	0.009041	0.156888	DLG2,GRIN2B,LRRC7
Negative regulation of the PI3K/AKT network	Signal Transduction	0.010088	0.177288	ERBB3,ERBB4,ESR1,FGFR2,NRG1,NRG2,NRG3
Ca2+ pathway	Signal Transduction	0.011461	0.177288	ITPR2,LEF1,NFATC1,TCF7L2,TNRC6B
Protein-protein interactions at synapses	Neuronal System	0.012109	0.177288	DLG2,EPB41L2,GRIN2B,NLGN1,NRXN3,PTPRD
Nef and signal transduction	Disease	0.012320	0.177288	DOCK2,ELMO1
Binding of TCF/LEF:CTNNB1 to target gene promoters	Signal Transduction	0.012320	0.177288	LEF1,TCF7L2
Phase 3 - rapid repolarisation	Muscle contraction	0.012320	0.177288	KCNE4,KCNQ1
RUNX3 regulates WNT signaling	Gene expression (Transcription)	0.012320	0.177288	LEF1,TCF7L2
Deactivation of the beta-catenin transactivating complex	Signal Transduction	0.013199	0.181103	LEF1,MEN1,TCF7L2,TLE4
tRNA Aminoacylation	Metabolism of proteins	0.013199	0.181103	FARS2,IARS,LARS2,WARS
Cell-cell junction organization	Cell-Cell communication	0.013879	0.186105	CDH13,CDH4,CTNND1,INADL,PARD6B
Voltage gated Potassium channels	Neuronal System	0.014308	0.187594	KCNA2,KCNQ1,KCNQ3,KCNS3
CHL1 interactions	Developmental Biology	0.015612	0.200241	ITGB1,NRP1
Phase 2 - plateau phase	Muscle contraction	0.016832	0.211295	CACNA2D3,KCNE4,KCNQ1
Caspase activation via Dependence Receptors in the absence of ligand	Programmed Cell Death	0.019235	0.226973	DAPK1,DCC
Regulation of commissural axon pathfinding by SLIT and ROBO	Developmental Biology	0.019235	0.226973	DCC,ROBO2
Diseases of DNA repair	Disease	0.019235	0.226973	MSH3,MSH6
CREB1 phosphorylation through NMDA receptor-mediated activation of RAS signaling	Neuronal System	0.020732	0.239911	DLG2,GRIN2B,LRRC7
Signaling by FGFR2 amplification mutants	Disease	0.021958	0.239911	FGFR2
Defective CYP19A1 causes Aromatase excess syndrome (AEXS)	Disease	0.021958	0.239911	CYP19A1
Signaling by FGFR2 fusions	Disease	0.021958	0.239911	FGFR2
CLEC7A (Dectin-1) induces NFAT activation	Immune System	0.023174	0.246904	ITPR2,NFATC1
RAF/MAP kinase cascade	Signal Transduction;Immune System	0.023435	0.246904	DLG2,ERBB3,ERBB4,FGFR2,GRIN2B,LRRC7,NF1,NRG1,NRG2,NRG3,TEK
Intracellular signaling by second messengers	Signal Transduction	0.024979	0.258555	ERBB3,ERBB4,ESR1,FGFR2,ITPR2,NRG1,NRG2,NRG3,PHC1,PPARG,PREX2,PRKCE,TNRC6B
MAPK1/MAPK3 signaling	Signal Transduction	0.026242	0.266944	DLG2,ERBB3,ERBB4,FGFR2,GRIN2B,LRRC7,NF1,NRG1,NRG2,NRG3,TEK
NOTCH2 intracellular domain regulates transcription	Signal Transduction	0.027411	0.269757	FCER2,MAML2
SUMOylation of intracellular receptors	Metabolism of proteins	0.027433	0.269758	ESR1,PPARG,RORA
Stimuli-sensing channels	Transport of small molecules	0.029125	0.281701	ANO3,ANO4,ANO6,ASIC2,NALCN,UNC80
Post NMDA receptor activation events	Neuronal System	0.029660	0.282248	DLG2,ERBB4,GRIN2B,LRRC7,NRG1
Adherens junctions interactions	Cell-Cell communication	0.035149	0.329173	CDH13,CDH4,CTNND1
MAPK family signaling cascades	Signal Transduction	0.039274	0.362057	DLG2,ERBB3,ERBB4,FGFR2,GRIN2B,LRRC7,

				NF1,NRG1,NRG2,NRG3,TEK,TNRC6B
Beta-catenin independent WNT signaling	Signal Transduction	0.040650	0.366112	DAAM1,ITPR2,LEF1,NFATC1,SMURF1,TCF7L2,TNRC6B
Regulation of gene expression in late stage (branching morphogenesis) pancreatic bud precursor cells	Developmental Biology	0.041773	0.366112	MAML2,ONECUT3
Mismatch Repair	DNA Repair	0.041773	0.366112	MSH3,MSH6
Defective Mismatch Repair Associated With MSH3	Disease	0.043437	0.366112	MSH3
Defective Mismatch Repair Associated With MSH6	Disease	0.043437	0.366112	MSH6
Defective ALG14 causes congenital myasthenic syndrome (ALG14-CMS)	Disease	0.043437	0.366112	ALG14
Formation of the beta-catenin:TCF transactivating complex	Signal Transduction	0.044702	0.371467	LEF1,MEN1,TCF7L2,TLE4
L1CAM interactions	Developmental Biology	0.047048	0.385532	ANK3,CNTNAP1,DNM3,ITGB1,KCNQ3,NRP1
PIP3 activates AKT signaling	Signal Transduction	0.049644	0.395810	ERBB3,ERBB4,ESR1,FGFR2,NRG1,NRG2,NRG3,PHC1,PPARG,PREX2,TNRC6B
FLT3 Signaling	Immune System	0.049644	0.395810	DLG2,ERBB3,ERBB4,FGFR2,GRIN2B,LRRC7,NF1,NRG1,NRG2,NRG3,TEK

The 74 additional pathways identified by the best performing probabilistic/additive machine learning model (multinomial naive Bayes model in dataset 4), ranked by p-value. The second rightmost columns shows the p-value corrected for a false discovery rate of 0.1 using the Bnjamini-Hochberg method. Biologically relevant genes from analyzed dataset are reported for each pathway. FDR: false discovery rate..
